# Scalable, trustworthy generative model for virtual multi-staining from H&E whole slide images

**DOI:** 10.1371/journal.pcbi.1013516

**Published:** 2025-10-21

**Authors:** Mehdi Ounissi, Ilias Sarbout, Jean-Pierre Hugot, Christine Martinez-Vinson, Dominique Berrebi, Daniel Racoceanu

**Affiliations:** 1 Sorbonne Université; CNRS; Inserm; Inria; Paris Brain Institute – ICM; AP-HP, Paris, France; 2 Paris Cité University; AP-HP; Centre de Recherche sur l’inflammation, Paris, France; 3 AP-HP; Robert Debré University Hospitals; Centre de Recherche sur l’inflammation, Paris, France; 4 Paris Cité University; Inserm; AP-HP; Necker-Enfants Malades University Hospitals; Anatomopathology Department; Centre de recherche sur l’inflammation, Paris, France; Universita degli Studi di Torino, ITALY

## Abstract

Chemical staining methods, while reliable, are time consuming and can be resource-intensive, involving costly chemical reagents and raising environmental concerns. This underscores the compelling need for alternative solutions such as virtual staining, which not only accelerates the diagnostic process but also enhances the flexibility of stain applications without the associated physical and chemical costs. Generative artificial intelligence technologies prove to be immensely useful in addressing these challenges. However, in healthcare, particularly within computational pathology, the high-stakes nature of decisions complicates the adoption of these tools due to their often opaque processes. Our work introduces an innovative approach that harnesses generative models for virtual stain transformations, improving performance, trustworthiness, scalability, and adaptability within computational pathology. The core of the proposed methodology involves a singular Hematoxylin and Eosin (H&E) encoder that supports multiple stain decoders. This design prioritizes critical regions in the latent space of H&E tissues, leading to a richer representation that enables precise synthetic stain generation by the decoders. Tested to simultaneously generate eight different stains from a single H&E slide, our method also offers significant scalability benefits for routine use by loading only necessary model components during production. We integrate label-free knowledge during training, using loss functions and regularization to minimize artifacts, thereby enhancing the accuracy of virtual staining in both paired and unpaired settings. To build trust in these synthetic stains, we employ a real-time self-inspection methodology using trained discriminators for each stain type, providing pathologists with confidence heatmaps to aid in their evaluations. In addition, we perform automatic quality checks on new H&E slides to ensure that they conform to the trained H&E distribution, guaranteeing the generation of high-quality synthetic stained slides. Recognizing the challenges pathologists face in adopting new technologies, we have encapsulated our method in an open-source, cloud-based proof-of-concept system. This system enables users to easily and virtually stain their H&E slides through a browser, eliminating the need for specialized technical knowledge and addressing common hardware and software challenges. It also facilitates real-time user feedback integration. Lastly, we have curated a novel dataset comprising eight different paired H&E/stains related to pediatric Crohn’s disease at diagnosis, providing 30 whole slide images (WSIs) for each stain set (total of 480 WSIs) to stimulate further research in computational pathology.

## Author summary

We address the challenges of chemical staining in pathology, including high costs, environmental impact, and time consumption, by introducing a virtual staining approach powered by generative artificial intelligence. Our method uses a unified Hematoxylin and Eosin (H&E) encoder with multiple stain decoders, allowing accurate generation of up to eight distinct stains from a single H&E slide. By focusing on critical tissue regions in the latent space, we achieve highly precise synthetic stains while minimizing artifacts in both paired and unpaired scenarios. To enhance trust and usability, we include real-time confidence heatmaps and automatic quality checks, ensuring high-quality output and providing transparency for pathologists. We further simplify adoption by offering an open source, cloud-based tool that enables virtual staining through a browser without technical expertise. To support future research, we share a novel dataset of 480 whole slide images comprising eight stain types for pediatric Crohn’s disease. Our work combines innovation, scalability, and accessibility to advance computational pathology and reduce the reliance on traditional chemical staining methods.

## 1. Introduction

Well-recognized standard practice in histopathology, Hematoxylin and Eosin (H&E) staining offers multiple benefits, making it a traditionally preferred choice in histopathology, worldwide. Not only does this technique deliver efficient and cost-effective results, it has also firmly established its place within routine reference diagnostic protocols in anatomopathology, reaching an indisputable central role in diagnostic protocols, such as cancer classification [1,2]. Thus, quantitative and qualitative measures on H&E staining represent the reference for the design of treatment strategies.

Despite these advantages, an intrinsic limitation of H&E staining is represented by its restricted potential to identify specific proteins within cells, an endeavor often considered crucial for pinpoint diagnosis of disease and/or severity evaluation.

To compensate for this drawback, immunohistochemical staining (IHC) represents a widely accepted viable solution because it is particularly effective in identifying specific proteins within cells. This characteristic is crucial in the classification of various types of tumors and is a key for pinpointing the origin of metastatic tumors. In addition, it can reveal minute tumor cells that might escape detection through standard staining procedures. This technique is particularly beneficial for diagnosing diseases that traditional biopsy cultures and serological diagnoses struggle to detect [3,4].

Despite these strengths, IHC procedures have significant limitations. These methods require substantial resources, including time-consuming sample preparation and expert supervision, which increase the likelihood of errors and delays. Such delays could adversely affect the diagnosis and treatment of the disease. Furthermore, the toxicity of the chemicals used in IHC can compromise the further analysis of the same tissue samples and can pose environmental hazards [5].

The inherent shortcomings of both H&E and IHC staining techniques highlight the pressing need for an automated, digital, and reliable process, at least for the pre-selection of key stains from a wide array of possibilities. Such an enhanced process should aim to improve diagnostic precision while circumventing the associated time and monetary constraints.

In this context, the latest advances in computational pathology deserve special attention, particularly the utilization of deep learning methods. These methods can successfully convert H&E stains into other IHC stains [5]. Additionally, deep learning techniques have demonstrated the capacity to generate synthetic IHC based on H&E slides, a process that has been shown to enhance diagnostic accuracy [6].

Both supervised and unsupervised deep learning methods have demonstrated promising outcomes in transforming H&E to IHC stains across diverse organ types [7–9]. This transformation can be accomplished through two primary techniques: supervised translation, often termed *"paired"*, and unsupervised translation, termed *"unpaired"*.

The paired translation method utilizes both H&E and other chemically stained slides for transformation. This may involve using the same H&E slide after washing and re-staining or using an adjacent slice. In contrast, the unpaired translation method does not require specific alignment between H&E and stained slides. Together, these techniques demonstrate significant potential to advance the field of computational pathology.

However, trust issues associated with existing methodologies, particularly deep generative models, remain a significant barrier. Clinicians and pathologists frequently find it difficult to rely on the predictions produced by these models, especially in real-world applications. Furthermore, the requirement for specialized hardware and software adds to the complexity. These technical requirements often pose challenges in seamlessly integrating computational techniques into routine pathology procedures.

We introduce a novel computational pathology pipeline that enhances the scalability, accuracy, trustworthiness, and utility of virtual staining techniques. Our approach includes a unified encoder that serves multiple stain decoders, trust-building mechanisms through self-inspection, advanced training methodologies without additional annotations, cloud-based deployment, and a unique dataset focused on pediatric Crohn’s disease. The distinguishing features of our research are structured around the next key contributions:

1. **Unified H&E encoder for multiple stain decoders:** We introduce a single, shared H&E encoder that efficiently serves multiple decoders for generating diverse synthetic stains. This innovative architecture improves learning capabilities and scalability by eliminating the need for multiple encoders. Extensive validation shows that our encoder can support up to eight distinct decoders simultaneously, significantly improving system performance and precision in synthetic stain generation.
2. **Annotation-free, knowledge-guided training via loss functions and regularization:** Our methodology advances the training of H&E encoders using specialized loss functions that do not require additional annotations. These functions use existing stain data as a reference, penalizing inaccuracies more severely and enhancing model reliability. We have also tailored our approach for both paired and unpaired staining scenarios, ensuring accurate stain transformations across diverse conditions.
3. **Trust enhancement in virtual stains through self-inspection and XAI heatmaps:** We prioritize reliability in synthetic stains with a dual mechanism of self-inspection and explainable AI (XAI). Our system uses discriminator models to assess the alignment of input slides with training data, as well as heatmaps for real-time feedback, highlighting discrepancies in stain quality and fidelity. This approach empowers pathologists with tools for better decision-making and strengthens the trustworthiness of synthetic output.
4. **Cloud-based virtual staining system:** We design, develop, and deploy a proof-of-concept of our virtual staining method on an open-source cloud-based platform. This innovative system allows pathologists to upload whole slide images (WSIs), generate synthetic stains, and provide feedback remotely. By eliminating the need for specialized hardware and software, this approach simplifies the use and accelerates the adoption of advanced staining technologies, providing guidelines that make them more accessible and user-friendly.
5. **Novel H&E/IHC paired stains dataset for pediatric Crohn’s disease:** We curated a unique dataset of paired H&E/special stains specific to pediatric Crohn’s disease. This dataset, comprising 30 slides per stain type (480 slides in total), is designed to foster further research and development in the field.

## 2. Methods

### Ethics statement

This study has been approved by the Institut National de la Santé et de la Recherche Médicale (INSERM) ethics committee (Institutional Review Board IRB00003888, approval reference 21-761) in March 2021. This study was conducted in accordance with the principles of the Declaration of Helsinki and all patients or their legal representatives (for minors) provided written informed consent.

### 2.1. Related works

Virtual staining has been proposed to enable efficient transformations between different types of stains for WSIs. In recent years, several improvements have been made in generating multiple stains using adversarial generative networks. However, substantial issues remain regarding scalability, accuracy, trustworthiness, and accessibility to clinicians [5,10]. This section explores the existing body of literature with an emphasis on the H&E to IHC transformation process, aiming to highlight these current limitations and provide a comprehensive understanding of the landscape in which our research is located.

#### 2.1.1. Stain synthesis using deep learning.

The field of computational pathology extensively investigates stain transformations and synthesis. These studies aim to accurately digitally emulate tissue slide staining using paired datasets, in which both H&E stain and the corresponding WSI in other stains are included. There are several noteworthy studies in this field. [6] used a deep learning model that simultaneously processes H&E tiles and outputs Jones, MT, and PAS stains. Similarly, [11] developed the SHIFT method from a paired pancreas dataset to convert H&E into virtual immunofluorescence images and estimate the distribution of the pancytokeratin tumor cell marker. Based on this, [12] used a dataset of paired gastric carcinomas dataset to generate cytokeratin staining from H&E to assist in the diagnosis of gastric cancer. [13] applied a paired prostate dataset to transform H&E to CK8 IHC stains, a preliminary step in reconstructing 3D segmented glands for stratification of prostate cancer risk. [14] also developed a pyramid approach to generate human epidermal growth factor receptor 2 IHC stain from H&E by using a paired breast cancer dataset.

Despite these advancements, the paired H&E/IHC staining method has some significant drawbacks. [15] highlights that staining processes are typically irreversible and present logistical and technical difficulties when acquiring paired data. In addition, inconsistencies in one type of stain can compromise the accuracy of the other, reducing the overall diagnostic value.

To address these limitations, alternative approaches are needed. An example is the C-DNN [15] method using cascaded deep neural networks to transform images from auto-fluorescence to H&E, then to PAS, circumventing the challenge of acquiring paired data. Moreover, unpaired dataset settings have also been explored with the CycleGAN [16,17], a model widely employed. Unpaired datasets have been used in studies such as [18] to transform H&E to trichrome and [19] to convert H&E and PHH3 stains. Other works include [20], which used a perceptual embedding consistency loss in GANs, and [21], which generated Ki-67-stained images from H&E-stained images. Furthermore, the MVFStain framework [22] was able to convert H&E-stained images into multiple virtual functional stains in various scenarios.

In the literature, two principal methodological approaches for domain representation are distinguished. The first strategy employs separate pairs of encoders, decoders, and discriminators for each domain pairing, as exemplified by CycleGAN [17] and its variants. This approach requires training of 3×n models, often resulting in scalability challenges during the training phase due to the extensive computational resources required. In contrast, methods such as StarGAN [23,24] utilize a unified model comprising a mapping network, a style encoder, a generator, and a discriminator. This configuration allows the generation of multiple latent domain representations, styled as distinct domains, which simplifies the training process by using a single model to accommodate multiple transformations.

However, these methodologies exhibit certain limitations, particularly in specialized applications. For example, a pathologist who requires a subset of stains - specifically *s* out of *S* potential stains created using H&E—faces a substantial computational burden. They need to either load 2×s models (an encoder and a decoder for each required stain) or use an overarching model that encompasses all *S* stains. Both options require considerable computational power, thus delaying critical real-time responses. Furthermore, the current scope of virtual staining technology does not support the simultaneous synthesis of more than three IHC stains in a single session, a significant limitation when it comes to scaling and meeting the diverse needs of clinical environments.

Despite these challenges in digital pathology, the broader field of image processing has seen notable progress in addressing similar scalability issues. For instance, in the realm of unpaired art-style transfers, approaches such as those presented in [25] employ a separate encoder, decoder, and discriminator for each domain, demonstrating substantial scalability potential. This success in other fields suggests that similar methodologies could be adapted for digital pathology, potentially enhancing scalability and efficiency in a domain where they are sorely needed.

The evolution of computational pathology has significantly benefited from diverse training strategies, loss functions, and regularization techniques. Work such as [26,27] has contributed to substantial improvements in model performance. However, embedding knowledge in a self-supervised manner without relying on additional labels continues to pose a complex challenge.

Moreover, the context within which computational pathology operates, particularly the selection of magnification in WSI interpretation, has gained increasing attention. Studies such as [28–30] have demonstrated the critical role of context in enhancing the performance of deep learning models for tissue characterization and cell classification. However, in the realm of virtual staining, there remains a significant gap with methods that often rely on arbitrary magnification scale choices. This underscores the importance of further exploration in virtual staining techniques that utilize paired and unpaired datasets, with the aim of improving their applicability and effectiveness.

In conclusion, the profound influence of synthetic stains on patient outcomes requires these methods to be both efficient and trustworthy. Concerns related to their interoperability and consistency are pressing areas for improvement, which our work aims to address in the existing academic landscape.

#### 2.1.2. Virtual staining public datasets.

In computational pathology, stain transformations and synthesis are pivotal research areas aimed at improving diagnostic accuracy. Many of these studies are based on the use of diverse datasets. Several of them employ paired datasets, which comprise both H&E stains and their corresponding IHC stains on the same tissue slide. For example, a notable study by [11] used a paired dataset of pancreatic tissues to develop the SHIFT method, which transforms H&E images into virtual PanCK immunofluorescence images, thus estimating the distribution of the tumor cell marker pancytokeratin. In a similar vein, [6] used a dataset of paired tissue slides to transform H&E tiles into Jones, Masson’s Trichrome and Periodic Acid-Schiff stains.

In addition, research has been extended to datasets that feature various types of cancers. [12] used a paired dataset of gastric carcinomas to produce cytokeratin staining from H&E, assisting in diagnosing gastric cancer. [13] employed a paired prostate dataset for transforming H&E to CK8 IHC stains, with the aim of reconstructing 3D segmented glands for stratification of prostate cancer risk. Meanwhile, [14] focused on generating human epidermal growth factor receptor 2 (HER2) IHC stain from H&E using a paired breast cancer dataset.

However, while paired datasets are instrumental, they are not without challenges. Since staining procedures are generally irreversible, acquiring such data can be technically challenging [15]. Recognizing these challenges, researchers have begun to explore unpaired datasets. For example, [18] successfully transformed H&E to trichrome using an unpaired liver dataset and used a skin and lymph node dataset to change H&E to SOX10 IHC. In another innovation, [20] applied a perceptual embedding consistency loss in GANs, leveraging an unpaired liver dataset to morph H&E into FAP-CK IHC stain. Beyond this, studies such as [21] have produced Ki-67-stained images from H&E-stained ones using unpaired and unbalanced datasets from neuroendocrine tumors and breast cancers. The MVFStain [22] framework is also notable, transforming H&E-stained images into various virtual functional stains for tissues such as mouse lung, breast cancer, and rabbit cardiovascular system.

However, even with these strides in computational pathology, data availability remains a bottleneck, especially the scarce good quality public availability of paired H&E/IHC stain datasets. To illustrate, although [6] publicly shared their approach’s source code, the dataset they utilized remains private. Moreover, specific domains, such as pediatric Crohn’s disease at the diagnosis stage (pretreatment), remain under-researched and present opportunities for future exploration.

#### 2.1.3. Cloud-based computational pathology.

In recent years, the landscape of collaborative image analysis systems has witnessed significant advances. A series of influential platforms emerged to reshape the domain. QuPath [31] introduced the concept of remote web-based collaboration to computational pathology, allowing for annotation and the addition of modulable algorithms via Javascript and Groovy. In addition, the Open Reproducible Biomedical Image Toolkit (ORBIT) [32] was launched, specializing in the orchestration of existing analysis tools for medical imaging. Its collaborative capacities were enhanced by integrating OMERO [33,34].

Despite limited AI capabilities in some tools, Cytomine [35] differentiates itself with its innovative web-based interface. It was the first platform to enable the display of multiple WSIs in a web environment, eliminating the need for software installation. Cytomine’s platform is notably comprehensive, incorporating all essential elements for server deployment, including web servers, job concurrency management, data storage, and a robust API. This integration makes it particularly suitable for histopathological applications.

Moreover, it enhances inclusivity and the reproducibility of results by supporting any dockerized algorithm. This feature allows authorized users access to a wide range of tools for collaborative medical image analysis. The platform’s design facilitates job monitoring and enhances user interaction, which in turn improves collaboration and workflow management. Due to its effectiveness in promoting collaboration, managing medical image data efficiently, and integrating advanced machine learning techniques, it is increasingly favored for various applications.

To the best of our knowledge, no cloud-based open-source platform has previously incorporated virtual staining in a reliable manner. In response to this gap and in alignment with our technological advances and research objectives, we have integrated our virtual staining method into the platform as a proof-of-concept. This integration provides a framework that empowers pathologists by eliminating the need for specific hardware and software requirementsthereby saving time and improving diagnostic and research capabilities. This is achieved through a browser interface, where all complex computations are managed in the backend, streamlining the user experience.

### 2.2. Data

In this study, we introduce a rigorously curated dataset that is crucial for our research. This dataset was acquired at Robert Debré Hospital in Paris within a study on pediatric Crohn’s disease.

**Population:** The study focuses on pediatric and adult patients diagnosed with Crohn’s disease according to the ESPGHAN criteria (European Society for Pediatric Gastroenterology Hepatology and Nutrition). These patients were followed at Robert Debré Hospital for at least one year and had an initial biopsy at the time of diagnosis. The study includes all patients diagnosed at the center from 1988 to 2019. Patients for whom whole slides were too old were removed, resulting in one or multiple slides available for 59 patients. This population includes predominantly male individuals (69%) and has a mean age of 11.11 years (standard deviation 3.64).

**Dataset description:** The dataset comprises a total of 480 whole-slide images (WSIs), evenly distributed across eight paired combinations of H&E and IHC stains. Each combination includes 30 matched pairs of an H&E slide and its IHC-stained counterpart, featuring the following markers: Anticytokeratin AE1/AE3 (AE1AE3), CD117 (c-Kit), CD15 (Lewis X or SSEA-1), CD163 (macrophage marker), CD3 (T cell co-receptor), CD8 (T cell co-receptor), cluster D2-40 (D240), and Giemsa stain. These 480 slides were derived from 59 patients, with 30 matched pairs (H&E and IHC from the same tissue region) available. All slides were scanned at a uniform magnification of 40× using the same scanner, with a resolution of 0.22 μm per pixel to ensure consistency.

For the experiments, the dataset was randomly divided into two subsets: 20% for testing and 80% for training. This split was performed at the slide level and considered the amount of tissue per slide, ensuring that each slide contained at least 10% tissue coverage (see S1 Text for exact slide IDs used for testing). Both the paired and unpaired training settings utilized the same total number of WSIs. In the paired setting, the data loader ensured a strict correspondence between an H&E slide and its IHC counterpart from the same tissue region during training. Conversely, in the unpaired setting, no such correspondence was maintained, and the slides were presented independently. Since the dataset itself remained unchanged, any observed performance differences arose solely from how the data was structured and presented to the model. The unpaired setting introduced a form of data augmentation by allowing the model greater flexibility, potentially reducing biases inherent to tissue-specific staining patterns and contributing to the improved performance observed in this setting.

Additionally, for S8 Fig, we used a separate in-house dataset comprising H&E-stained slides only. This dataset, which cannot be made publicly available due to privacy concerns, consists of a cohort of pediatric patients diagnosed with Crohn’s disease and followed at Robert Debré Hospital in Paris, France. The biological material included paraffin-embedded tissue blocks obtained from diagnostic gastrointestinal biopsies collected as part of routine clinical care. A total of 2022 slides were available for analysis. Each slide (5 μm thick) was stained with H&E to obtain high-quality sections suitable for scanning. All slides were digitized using a Hamamatsu slide scanner at the pathology department of AP-HP and were semi-anonymized prior to analysis.

### 2.3. Multi virtual staining model architecture and training methodology

Our research focuses mainly on adapting two notable neural network architectures, ComboGAN [25] and CycleGAN [17], for a specific application: transforming H&E-stained histological slides into various other types of stain. The core architectural framework of our approach includes key components such as an encoder *E*_*i*_, a generator *G*_*i*_, and a discriminator *D*_*i*_, where *i* denotes the index representing each unique stain type within the set {1,..., S}. A crucial contribution of our method is the utilization of shared (unique) H&E-specific encoder, generator, and discriminator in all *S* stains, denoted as $E_{\text{H\&E}}$, $G_{\text{H\&E}}$, and $D_{\text{H\&E}}$, respectively. This strategic choice aims to improve the efficacy and specificity of the transformation process.

**Synthesis training:** The training methodology we employ is based on a dual cycle process aimed at transforming and reconstructing stains in histopathology slides. This approach focuses on seamlessly converting between H&E stained tiles and various other stain types, while ensuring preservation of core structural features throughout transformations.

In the first cycle of this process "H&E cycle", we start with an H&E-stained tile (refer to S5A Fig). This tile is first processed by an encoder specific to H&E stains, denoted as $E_{\text{H\&E}}$, transforming it into a latent representation $Z_{\text{H\&E}}$. This latent representation is then fed into a generator *G*_*i*_, corresponding to the type *i* of target stain, producing an image ${\hat{Y}}_{i}$ that mirrors the characteristics of the desired stain. To complete the cycle, this generated image is passed through another encoder *E*_*i*_, obtaining a new latent representation ${\hat{Z}}_{\text{H\&E}}$, which is subsequently used by the H&E generator $G_{\text{H\&E}}$ to recreate an H&E-stained tile ${\hat{X}}_{\text{H\&E}}$. Mathematically, this reconversion process, which ensures the cycle from an H&E stain to a target stain *i* and back to H&E, can be encapsulated as follows:

$${\hat{Y}}_{i}=G_{i}\left(E_{\text{H\&E}}\left(X_{\text{H\&E}}\right)\right),$$ (1)

$${\hat{X}}_{\text{H\&E}}=G_{\text{H\&E}}\left(E_{i}\left({\hat{Y}}_{i}\right)\right)\;\forall i\in \{1,\ldots ,S\}$$ (2)

In the second cycle "stain *i* cycle", we address the reverse process: starting with a tile originally stained with a specific type *i*, our objective is to convert it into an H&E-stained representation and subsequently revert it to its original stain (refer to S5B Fig). Initially, tile *X*_*i*_ undergoes encoding via *E*_*i*_ to produce a latent representation *Z*_*i*_. This is then converted into ${\hat{Y}}_{\text{H\&E}}$ an H&E-stained tile by the H&E generator $G_{\text{H\&E}}$, effectively translating *X*_*i*_ into the H&E domain. The resulting H&E image is re-encoded by $E_{\text{H\&E}}$ into a new latent representation ${\hat{Z}}_{i}$, which serves as input for the generator *G*_*i*_, reconstructing the original stained image ${\hat{X}}_{i}$. This enables the conversion of any specific stain type to an H&E representation and back, formulated mathematically as:

$${\hat{Y}}_{\text{H\&E}}=G_{\text{H\&E}}\left(E_{i}\left(X_{i}\right)\right),$$ (3)

$${\hat{X}}_{i}=G_{i}\left(E_{\text{H\&E}}\left({\hat{Y}}_{\text{H\&E}}\right)\right)\;\forall i\in \{1,\ldots ,S\}$$ (4)

To effectively train our architecture, we introduce a comprehensive global synthesis loss, which we denote as ${ℒ}_{\text{IHC},i}$. This loss facilitates the translation from H&E stained images to a specific target stain, represented by *i*. The process involves comparing the reconstructed image ${\hat{X}}_{i}$ and the target image *X*_*i*_ for the specified stain *i*, as well as comparing the reconstructed H&E image ${\hat{X}}_{\text{H\&E}}$ and the original H&E image $X_{\text{H\&E}}$. These comparisons are used to estimate the cycle loss for each stain *i*, denoted as ${ℒ}_{\text{cyc},i}$, which quantifies the fidelity of translation between the H&E stain and stain *i* in a bidirectional manner.

In addition to the cycle loss, our model also incorporates an adversarial loss, ${ℒ}_{\text{adv},i}$. This component utilizes discriminators in inference, specifically $D_{\text{H\&E}}$ for the H&E stain and *D*_*i*_ for the target stain *i*, to assess the authenticity of the generated images ${\hat{Y}}_{i}$ and ${\hat{Y}}_{\text{H\&E}}$. The discriminators aim to distinguish between real and synthesized images, thus encouraging the generation of images that are indistinguishable from genuine stained samples. Furthermore, the model incorporates a regularization loss, ${ℒ}_{\text{reg},i}$, which helps the model converge. The overall synthesis loss function for each specific stain *i*, out of a total of *S* stains, is given by the equation:

$${ℒ}_{\text{IHC},i}=\lambda_{\text{cyc}}\cdot {ℒ}_{\text{cyc},i}+\lambda_{\text{adv}}\cdot {ℒ}_{\text{adv},i}+\lambda_{\text{reg}}\cdot {ℒ}_{\text{reg},i}\;\forall i\in \{1,...,S\}$$ (5)

Here, $\lambda_{\text{cyc}}$, $\lambda_{\text{adv}}$ and $\lambda_{\text{reg}}$ are weighting coefficients. This approach ensures a versatile and effective training regime that can accommodate a range of stains, denoted by *S*, improving the model’s ability to generalize across a wide range of stains.

**Discriminator training:** In our approach, we employ two types of discriminators: one for the H&E stains, denoted as $D_{\text{H\&E}}$, and individual discriminators for each IHC stain, represented as *D*_*i*_ for the *i^th^* stain. Each discriminator is trained using its respective loss function to optimize its performance.

For the H&E discriminator, $D_{\text{H\&E}}$, the loss function is defined as follows:

$${ℒ}_{D_{\text{H\&E}}}=\lambda_{\text{D}}\cdot ({ℒ}_{{\text{real}}_{\text{H\&E}}}+{ℒ}_{{\text{synthetic}}_{\text{H\&E}}})$$ (6)

This equation represents a combination of the losses ${ℒ}_{{\text{real}}_{\text{H\&E}}}$ and ${ℒ}_{{\text{synthetic}}_{\text{H\&E}}}$ from real and synthetic H&E stained images respectively (refer to Eq (15)), scaled by a weighting factor $\lambda_{\text{D}}$, to measure the discriminator’s performance in distinguishing between genuine and artificially generated H&E images.

Similarly, for each stain discriminator *D*_*i*_, the loss function is customized to assess its ability to discern real images from synthetic stained images of that particular type, defined as:

$${ℒ}_{D_{\text{IHC},i}}=\lambda_{\text{D}}\cdot ({ℒ}_{\text{real},i}+{ℒ}_{\text{synthetic},i})\;\forall i\in \{1,\ldots ,S\}$$ (7)

Here, ${ℒ}_{\text{real},i}$ and ${ℒ}_{\text{synthetic},i}$ correspond to the losses from real and synthetic images of the *i^th^* stain, respectively (refer to Eq (14)). The sum of these losses, weighted by $\lambda_{\text{D}}$, constitutes the total loss for that discriminator, ensuring that it effectively learns to differentiate between actual and generated samples of its specific stain. This structure is applied in all *S* stains, allowing discriminators to specialize in their respective stains for improved performance in identifying authentic images versus generated images.

#### 2.3.1. Integrating annotation-free knowledge through loss function optimization.

In the development of computational models for synthesizing IHC slides, it has become evident that traditional distances such as ${ℒ}_{1}$, ${ℒ}_{2}$, and mean square error (MSE) pose significant challenges. A key issue with these metrics lies in their indiscriminate treatment of different regions on the slides, as they fail to differentiate between tissue sections and areas activated by IHC staining. This becomes particularly problematic due to the inherent staining imbalance in IHC slides, where the vast majority of the slide is IHC-negative, underscoring that IHC staining is specific to only a small fraction of the total slide content.

To address these shortcomings, our approach harnesses the cycle consistency loss ${ℒ}_{\text{cyc}}$ and adversarial loss ${ℒ}_{\text{adv}}$ [17,25]. Building on these foundations, we propose a novel method that integrates IHC-activated areas into the model’s training process for synthesizing *S* stains from H&E stained slides. This strategy facilitates the extraction of annotation-free knowledge, seamlessly incorporating the distinctive characteristics of each stain into the model. Consequently, our model can generate features with enhanced fidelity, thus improving the overall quality of synthesis. By guiding the synthesis process with stain-specific insights, our method not only enhances performance but also the transparency and trustworthiness of the synthesized images.

To automate the extraction of a precise mask for the IHC-activated region, denoted *M*_*i*_, from the *i^th^* IHC-stained image, *X*_*i*_, we begin by transforming *X*_*i*_ from its original RGB color space to the HSV color space. This conversion is crucial because it significantly enhances the ability to isolate the target regions based on their color and brightness attributes. After converting the image to the HSV color space, we apply a threshold to isolate a distinct mask, *M*_*i*_, which is shown in S2 Fig. This mask *M*_*i*_ is used to calculate the dynamic weighting factors, $\alpha_{i}$ and $\beta_{i}$, which are integrated into the computation of the loss function. These factors are defined as follows:

$$\alpha_{i}=\frac{{\bar{N}}_{i}}{N_{i}+{\bar{N}}_{i}},\;\beta_{i}=\frac{N_{i}}{N_{i}+{\bar{N}}_{i}}$$ (8)

In this equation, *N*_*i*_ represents the total number of pixels in the background, which corresponds to the IHC-activated regions, and ${\bar{N}}_{i}$ represents the total number of pixels in the background, or the non-activated IHC regions, of the *i^th^* IHC-stained image. This approach enables nuanced differentiation between the regions of interest and their background, facilitating more accurate analyses.

**Integrating knowledge during the synthesis training phase (cycle loss):** Integration of knowledge during the synthesis training phase involves a detailed process that utilizes both H&E stained tiles and IHC images from specific stains. This process is important in creating accurate high-quality synthetic images that mirror the unique characteristics of each stain.

In scenarios where direct pairs of H&E-stained images and IHC images are not available, an *unpaired setting* approach is used. This uses a specifically designed cycle loss, ${ℒ}_{\text{cyc},i}$, to facilitate synthesis in the absence of paired images. The cycle loss formula for the unpaired setting is as follows:

$${ℒ}_{\text{cyc},i}=ℒ({\hat{X}}_{i},X_{i})+ℒ({\hat{X}}_{\text{H\&E}},X_{\text{H\&E}})\;+\alpha_{i}\cdot ℒ(M_{i}⊙{\hat{X}}_{i},M_{i}⊙X_{i})+\beta_{i}\cdot ℒ({\bar{M}}_{i}⊙{\hat{X}}_{i},{\bar{M}}_{i}⊙X_{i})\;\forall i\in \{1,\ldots ,S\}$$ (9)

This approach meticulously focuses on the differentiation between IHC-activated regions (*M*_*i*_) and non-activated regions (${\bar{M}}_{i}$), ensuring the integrity of the synthesis process despite the absence of direct image correspondences (refer to S5 Fig).

In contrast, in *paired settings* where there are direct correspondences between H&E and IHC images exist, the cycle loss is formulated to ensure both the overall fidelity of the image reconstructions and the accurate replication of specific IHC-activated regions while taking advantage of the direct correspondence (see S4 Fig). The comprehensive equation for the paired setting is the following:

$${ℒ}_{\text{cyc},i}=ℒ({\hat{X}}_{i},X_{i})+ℒ({\hat{X}}_{\text{H\&E}},X_{\text{H\&E}})\;+\alpha_{i}\cdot [ℒ(M_{i}⊙{\hat{X}}_{i},M_{i}⊙X_{i})+ℒ(M_{i}⊙{\hat{X}}_{\text{H\&E}},M_{i}⊙X_{\text{H\&E}})]\;+\beta_{i}\cdot [ℒ({\bar{M}}_{i}⊙{\hat{X}}_{i},{\bar{M}}_{i}⊙X_{i})+ℒ({\bar{M}}_{i}⊙{\hat{X}}_{\text{H\&E}},{\bar{M}}_{i}⊙X_{\text{H\&E}})]\;\forall i\in \{1,\ldots ,S\}$$ (10)

Both settings employ dynamic weighting factors, $\alpha_{i}$ and $\beta_{i}$, determined by the ratio of IHC-activated regions to non-activated regions within each image. This careful consideration ensures that the model’s training prioritizes not only the overall accuracy of stain transformation but also the faithful replication of regions crucial for IHC analysis. Furthermore, the methodology incorporates masks *M*_*i*_ and ${\bar{M}}_{i}$ in the calculation of the cycle loss, enhancing the model’s capacity to embed the distinctive characteristics of each stain directly into its architecture. This meticulous approach facilitates the integration of annotation-free knowledge, resulting in the production of high-quality, reliable synthetic images that accurately reproduce the complexities of IHC staining.

**Integrating knowledge during the synthesis training phase (adversarial loss):** In the context of the unpaired setting, the adversarial loss, denoted ${ℒ}_{\text{adv},i}$, is calculated by evaluating the authenticity of the generated images ${\hat{Y}}_{i}$ and $\hat{H\&E}$ through discriminators *D*_*i*_ and $D_{\text{H\&E}}$, respectively. This process is enhanced by using the stain mask *M*_*i*_, which helps $D_{\text{H\&E}}$ focus on the areas activated by the IHC, as shown in S5 Fig. The formula for computing adversarial loss in unpaired settings is as follows:

$${ℒ}_{\text{adv},i}=ℒ(D_{i}({\hat{Y}}_{i}),1)\;+\alpha_{i}\cdot ℒ(M_{i}⊙D_{\text{H\&E}}({\hat{Y}}_{\text{H\&E}}),1)\;+\beta_{i}\cdot ℒ({\bar{M}}_{i}⊙D_{\text{H\&E}}({\hat{Y}}_{\text{H\&E}}),1)\;\forall i\in \{1,\ldots ,S\}$$ (11)

For paired setting, the approach mirrors that of the unpaired setting but with an added emphasis on the direct correspondence between the H&E stain and image *i*, as shown in S4 Fig. The computation of adversarial loss for paired settings incorporates this direct correspondence and is expressed by the following equation:

$${ℒ}_{\text{adv},i}=ℒ(D_{\text{H\&E}}({\hat{Y}}_{\text{H\&E}}),1)\;+\alpha_{i}\cdot [ℒ(M_{i}⊙D_{\text{H\&E}}({\hat{Y}}_{\text{H\&E}}),1)+ℒ(M_{i}⊙D_{i}({\hat{Y}}_{i}),1)]\;+\beta_{i}\cdot [ℒ({\bar{M}}_{i}⊙D_{\text{H\&E}}({\hat{Y}}_{\text{H\&E}}),1)+ℒ({\bar{M}}_{i}⊙D_{i}({\hat{Y}}_{i}),1)]\;\forall i\in \{1,\ldots ,S\}$$ (12)

**Direct supervision loss (paired setting):** In the paired setting of our framework, the synthesis loss is meticulously designed to include a variety of components, notably the supervised loss (${ℒ}_{\text{sup},i}$), alongside the cycle consistency loss (${ℒ}_{\text{cyc},i}$) as mentioned in Eq (10) and the adversarial loss (${ℒ}_{\text{adv},i}$) as detailed in Eq (12). Crucially, supervised loss establishes a direct connection between the H&E cycle and the specific stain cycle *i*. It does this by evaluating the fidelity of the generated stains ${\hat{Y}}_{i}$ and H&E images ${\hat{Y}}_{\text{H\&E}}$ against their actual counterparts (*X*_*i*_ and $X_{\text{H\&E}}$, respectively). Furthermore, it incorporates a common mask (*M*_*i*_), derived from *X*_*i*_, to concentrate the loss computation on relevant areas of the image. This ensures that the generated images maintain both structural and stylistic integrity in relation to the original samples. The formula for the supervised loss is articulated as follows:

$${ℒ}_{\text{sup},i}=ℒ({\hat{Y}}_{i},X_{i})+ℒ({\hat{Y}}_{\text{H\&E}},X_{\text{H\&E}})\;+\alpha_{i}\cdot [ℒ(M_{i}⊙{\hat{Y}}_{i},M_{i}⊙X_{i})+ℒ(M_{i}⊙{\hat{Y}}_{\text{H\&E}},M_{i}⊙X_{\text{H\&E}})]\;+\beta_{i}\cdot [ℒ({\bar{M}}_{i}⊙{\hat{Y}}_{i},{\bar{M}}_{i}⊙X_{i})+ℒ({\bar{M}}_{i}⊙{\hat{Y}}_{\text{H\&E}},{\bar{M}}_{i}⊙X_{\text{H\&E}})]\;\forall i\in \{1,\ldots ,S\}$$ (13)

**Integrating knowledge during the discriminator training phase:** To effectively train the discriminator *D*_*i*_, we employ authentic *X*_*i*_ samples to generate corresponding stain masks *M*_*i*_. These masks enable the discriminator to discern nuances within IHC-activated regions through the ${ℒ}_{{\text{real}}_{i}}$ loss function. Concurrently, the discriminator is trained to recognize synthetic images ${\hat{Y}}_{i}$ as inauthentic using the ${ℒ}_{{\text{synthetic}}_{i}}$ loss function. The formulation of both loss functions is as follows:

$$\left\{ \begin{array}{c} {ℒ}_{{\text{real}}_{i}}=ℒ(D_{i}(X_{i}),1)+\alpha_{i}\cdot ℒ(M_{i}⊙D_{i}(X_{i}),1)+\beta_{i}\cdot ℒ({\bar{M}}_{i}⊙D_{i}(X_{i}),1)\\ {ℒ}_{{\text{synthetic}}_{i}}=ℒ(D_{i}({\hat{Y}}_{i}),0) \end{array}\right.$$ (14)

In a similar vein, for the $D_{\text{H\&E}}$ discriminator, we define ${ℒ}_{{\text{real}}_{\text{H\&E}}}$ and ${ℒ}_{{\text{synthetic}}_{\text{H\&E}}}$ to, respectively, discern the authenticity of H&E stained images and identify synthetic counterparts. These loss functions are described as follows:

$$\left\{ \begin{array}{c} {ℒ}_{{\text{real}}_{\text{H\&E}}}=ℒ(D_{\text{H\&E}}(X_{\text{H\&E}}),1)\\ {ℒ}_{{\text{synthetic}}_{\text{H\&E}}}=ℒ(D_{\text{H\&E}}({\hat{Y}}_{\text{H\&E}}),0) \end{array}\right.$$ (15)

#### 2.3.2. Enhancing training using regularization.

**One H&E representation to rule all the staining modalities:** The main objective of our approach is to develop a universal H&E encoder and generator capable of handling all staining modalities used in histology. Traditional methods often face challenges due to the varying requirements for different stains during the training phase. Some stains are inherently more complex to replicate, leading to uneven learning progress and possible neglect of less dominant stains. To address this issue, we have implemented a novel regularization strategy. This approach ensures an even distribution of learning focus across all stains by the components of our H&E encoder, thereby minimizing bias towards any specific stain.

The regularization process involves a systematic selection in which the stains are randomly chosen from a complete set identified by numbers ranging from 1 to *S*, ensuring complete coverage. The updates are then applied to the encoder *E*_*i*_ and the generator *G*_*i*_ for each selected stain *i*, alongside the updates for the shared H&E components ($E_{\text{H\&E}}$ and $G_{\text{H\&E}}$), ensuring a fair representation of each stain throughout the training cycles. After cycling through all *S* stains in a random sequence, we calculate a mean synthetic loss across them using the equation:

$${ℒ}_{\text{H\&E}}=\frac{1}{S}\sum_{i=1}^{S}{ℒ}_{\text{IHC},i}$$ (16)

This calculated loss, ${ℒ}_{\text{H\&E}}$, specifically refines the H&E $E_{\text{H\&E}}$ and $G_{\text{H\&E}}$, marking the completion of a training iteration. This methodology ensures equal attention to each stain and significantly enhances the versatility of the model in various modalities. As a result, we achieve a more stable and scalable training process, thereby enhancing the model’s overall effectiveness in dealing with a wide range of staining modalities.

**Stain synthesis regularization:** We propose a comprehensive methodology designed to encapsulate the entire spectrum of considerations for regularization in virtual staining. By integrating critical knowledge through IHC-activated regions captured by the stain mask *M*_*i*_. This process involves the calculation of a regularized loss, denoted as ${ℒ}_{\text{reg},i}$, formulated as a weighted sum of three principal loss functions: identity (${ℒ}_{\text{idt,}i}$), latent (${ℒ}_{\text{lat,}i}$), and forward (${ℒ}_{\text{fwd,}i}$) losses.

For every stain index *i* within the set {1,...,S}, the regularized loss is precisely defined as:

$${ℒ}_{\text{reg,}i}=\lambda_{\text{idt}}\cdot {ℒ}_{\text{idt,}i}+\lambda_{\text{lat}}\cdot {ℒ}_{\text{lat,}i}+\lambda_{\text{fwd}}\cdot {ℒ}_{\text{fwd,}i}$$ (17)

Here, the coefficients $\lambda_{\text{idt}},\lambda_{\text{lat}},$ and $\lambda_{\text{fwd}}$ represent the respective weights of each loss component within the cumulative regularized loss framework.

Identity loss (${ℒ}_{\text{idt}}$) quantifies the deviation between the original and generated images, using the same domain encoder and generator within an autoencoder setup. This ensures that the encoder captures enough features to directly reproduce the input image. This concept is further extended to include *M*_*i*_ as follows:

$${ℒ}_{\text{idt},i}=\left\{ \begin{array}{ll} ℒ(G_{i}(E_{i}(X_{i})),X_{i})\;+\alpha_{i}\cdot ℒ(M_{i}⊙G_{i}(E_{i}(X_{i})),M_{i}⊙X_{i})\;+\beta_{i}\cdot ℒ({\bar{M}}_{i}⊙G_{i}(E_{i}(X_{i})),{\bar{M}}_{i}⊙X_{i})\;\forall i\in \{1,\ldots ,S\}, & \text{for stain }i\text{ image }X_{i},\\ ℒ(G_{\text{H\&E}}(E_{\text{H\&E}}(X_{\text{H\&E}})),X_{\text{H\&E}}) & \text{for H\&E image }X_{\text{H\&E}}. \end{array}\right.$$ (18)

The latent loss (${ℒ}_{\text{lat}}$) aims to mitigate the disparities within the latent space, specifically by capturing the variance between the latent representation and its reconstructed version. This facilitates the alignment of embeddings from both H&E and stain *i* encoders, incorporating IHC-activated regions as follows:

$${ℒ}_{\text{lat},i}=\left\{ \begin{array}{cc} ℒ({\hat{Z}}_{i},Z_{i})\;+\alpha_{i}\cdot ℒ(M_{i}⊙{\hat{Z}}_{i},M_{i}⊙Z_{i})\;+\beta_{i}\cdot ℒ({\bar{M}}_{i}⊙{\hat{Z}}_{i},{\bar{M}}_{i}⊙Z_{i})\;\forall i\in \{1,\ldots ,S\}, & \text{for stain }i\text{ embeddings }Z_{i}\text{ and }{\hat{Z}}_{i},\\ ℒ({\hat{Z}}_{\text{H\&E}},Z_{\text{H\&E}}) & \text{for H\&E embeddings }Z_{\text{H\&E}},{\hat{Z}}_{\text{H\&E}}. \end{array}\right.$$ (19)

Finally, the forward loss assesses the divergence between the degraded versions (represented using the lower case) of the original images ($X_{i},X_{\text{H\&E}}$) and their corresponding outputs (${\hat{Y}}_{\text{H\&E}},{\hat{Y}}_{i}$), specified as:

$${ℒ}_{\text{fwd},i}=\left\{ \begin{array}{cc} ℒ({\hat{y}}_{\text{H\&E}},x_{i})\;+\alpha_{i}\cdot ℒ(m_{i}⊙{\hat{y}}_{\text{H\&E}},m_{i}⊙x_{i})\;+\beta_{i}\cdot ℒ({\bar{m}}_{i}⊙{\hat{y}}_{\text{H\&E}},{\bar{m}}_{i}⊙x_{i})\;\forall i\in \{1,\ldots ,S\}, & \text{for stain }i,\\ ℒ({\hat{y}}_{i},x_{\text{H\&E}}) & \text{for H\&E}. \end{array}\right.$$ (20)

By incorporating three distinct loss functions (${ℒ}_{\text{idt},i}$, ${ℒ}_{\text{lat},i}$, and ${ℒ}_{\text{fwd},i}$) and leveraging knowledge from IHC-activated regions, this regularization approach opens up a wide range of opportunities for fine-tuning the handling of task-specific challenges. This method is particularly designed for unpaired settings, where it contributes significantly to the nuanced management of such tasks. In paired settings, these approaches do not offer substantial benefits due to the direct correspondences between the H&E and the *S* stains, which are already extensively addressed by supervised loss functions. However, the theoretical potential of integrating these regularization strategies ${ℒ}_{\text{reg, i}}$ suggests a broader applicability beyond their initial intended contexts.

Note that the degradation process involved downscaling the H&E- and IHC-stained images using an average pooling operation. Specifically, we applied PyTorch’s AvgPool2d(3, stride=2) function, which performs a two-dimensional average pooling with a kernel size of 3 and a stride of 2. This operation reduces the spatial dimensions of the images by computing the average value over each 3×3 region and moving the window by two pixels at a time, thereby generating a degraded version of the images.

### 2.4. Trust in virtual stains through self-inspection–anomaly detection

As described in Sect 2.3, our architecture incorporates encoder, decoder, and discriminator components inspired by CycleGAN [17] and ComboGAN [25]. Central to our methodology is the use of a PatchGAN discriminator [17,36,37], which is explicitly designed to differentiate between synthetic and authentic images. This discriminator features dual heads: one that focuses on luminance (emitting a confidence map *C*_*lum*_) and the other on RGB space (emitting a confidence map *C*_*rgb*_). Each map rates the authenticity of the image on a clamped scale from -1 (’anomaly’) to 1 (’authentic’), and both maps are resized to match the size of the input image.

To simplify the analysis for pathologists and reduce cognitive load, we combine these two maps into a single map by calculating the pixel-wise minimum of *C*_*lum*_ and *C*_*rgb*_, resulting in *C*_*all*_. This combined confidence map *C*_*all*_ is then normalized to a range of 0 to 1, where 0 indicates an anomaly and 1 indicates authenticity. This map can be used to calculate various metrics, such as the standard deviation shown in S8 Fig. Additionally, we apply a Jet-color map using OpenCV version 4.9.0 [38] to transform *C*_*all*_ into an 8-bit unsigned integer RGB confidence map, as illustrated in S9 Fig.

This approach provides comprehensive confidence maps that can identify a wide range of anomalies, as demonstrated in S8 and S9 Figs. Given that the same discriminator architecture is utilized in all stains, this methodology is applicable to all of them.

### 2.5. Tile-stitching for clean virtual staining WSI generation using a Hamming window-based approach.

To address the inevitable stitching artifacts encountered during the reconstruction of synthetic WSIs, we applied a tailored image processing approach. Central to our methodology was the use of a two-dimensional (2D) Hamming window [39,40], designed to smooth the transitions between adjacent image patches and mitigate edge effects.

The Hamming window, traditionally used in signal processing [39,40] to taper the signal edges, was adapted to two dimensions to suit the image patches. Each patch, representing a portion of the larger image, was processed through this window to ensure a gradual transition at the borders. With an overlap > 0, this was achieved by computing the outer product of a one-dimensional Hamming window with itself, thus creating a symmetric 2D window *w*(*x*,*y*) for a patch of size M×M is defined as:

$$w(x,y)=0.54-0.46\cos\left(\frac{2\pi x}{M-1}\right)\cdot \left(0.54-0.46\cos\left(\frac{2\pi y}{M-1}\right)\right)$$ (21)

where *x*,*y* range from 0 to *M*–1. This results in a 2D Hamming window, which reduces the pixel values towards the edges of each patch. Then, this window was applied across the three color channels of the image. Each image patch (across all RGB channels) was element-wise multiplied by this matrix, reducing the intensity at the peripheries and thereby softening the boundaries between stitched patches. This operation is described by the following equation:

$$P_{\text{weighted}}(x,y)=P(x,y)\cdot w(x,y)$$ (22)

Where *P*(*x*,*y*) is the original pixel value at coordinates (*x*,*y*) within the patch for a given color channel, and *w*(*x*,*y*) is the value from the 2D Hamming window at these coordinates.

After applying the Hamming window, the weighted patches were summed to form the complete WSI. In regions where patches overlapped, pixel values from multiple patches were combined. To ensure uniformity, the accumulated weights of the patches were recorded and used to normalize the pixel values in these overlapping areas. This normalization process was crucial for maintaining consistent intensity across the WSI, preventing visual discontinuities that could hinder the quality of the synthetically stained WSIs. This methodology can be applied to any tile-based virtual staining approach to reconstruct a clean WSI output.

The final processed image was saved in pyramidal TIFF format, suitable for high-quality WSI. The processing pipeline was implemented using Python, using libraries such as NumPy [41] v1.26.3 for numerical operations and PyVIPS [42] v2.2.2 for image handling, ensuring efficient memory usage and scalability.

### 2.6. Cloud-based platform

We use open-source Cytomine Community Edition Legacy 3.1.0 software to transform our virtual staining model into a web application. It operates based on a containerized architecture using Docker, which facilitates the creation and deployment of Cytomine applications through various modules (applications, web UI, databases, nginx proxy, jobs management, etc.). The core component for the implementation of deep learning-based applications is a *software* Docker container.

Our Python-based application performing virtual staining is itself Dockerized and uploaded to software_router, where it is transformed into a Singularity image. Our python-based application includes the code for virtual staining, as well as the code to import the input WSI and upload the output virtual stains in the database. For these two last tasks, we use the Cytomine Python API for communication between the *software* container and the image database container.

The inputs to this Python-based application are specified in a JSON descriptor, also uploaded to the *software* container, which is then transformed into a user-friendly web interface for users to select the input H&E WSI.

When the user launches the algorithm initiating the virtual staining, its execution is managed by a SLURM-based job scheduling system, which launches the Singularity image with the corresponding inputs. Upon completion, the generated stains could be visualized directly within the Web UI.

Cytomine is optimized for the display of multiple instances of aligned WSIs, allowing for simultaneous visualization of stains. This functionality significantly improves the ability to compare and analyze different staining results within a unified interface, providing a powerful tool for digital pathology and related research fields.

### 2.7. Experimental configurations

To ensure reproducibility, it is important to note that all the experiments conducted in this study utilized the same architecture for the encoder, decoder, and discriminator. The number of parameters was aligned with those specified in [25] and implemented using the PyTorch library (version 2.2.0 with CUDA v12.1 and cuDNN v8.902) [43]. All training sessions were performed using 2048 x 2048 tiles resized to 512 x 512 tiles (no overlap) from the Crohn’s dataset, as discussed in Sect 2.2, in either paired or unpaired settings. The models employed an Adam optimizer with parameters $\beta_{1}=0.5$ and $\beta_{2}=0.999$, and a batch size of 6. We used only random flip and random rotation (data augmentation strategies). Each training epoch contains 728 iterations. Each training was conducted on a single NVIDIA A100 80GB GPU. In this study, the cycle-consistency loss coefficient ($\lambda_{\text{cyc}}$) was set to 10, consistent with the original CycleGAN implementation, which demonstrated stable convergence using this value across multiple image-to-image translation tasks, including artistic style transfer [17]. Given its widespread validation in the literature, this hyperparameter was adopted without an extensive hyperparameter search. Nevertheless, optimizing $\lambda_{\text{cyc}}$ through a systematic strategy (e.g., grid search or Bayesian optimization) may further improve model performance in this specific application and represents a potential direction for future work.

#### 2.7.1. Enhanced performance and efficiency in multi virtual staining using unified H&E encoder.

In S1 Table, we trained two different approaches — our unified method and CycleGAN (refer to S3 Fig)—. For CycleGAN, a separate model was trained for H&E to each of the different stains, with a total of eight stains. This involved 16 encoders, 16 decoders, and 16 discriminators. Each model underwent 75 epochs at a fixed learning rate of $2\;\times \;10^{-4}$, followed by 75 decay epochs with a linearly decaying rate, totaling 150 epochs per stain (1200 epochs overall). The loss weights were set at $\lambda_{\text{cyc}}=10$ and $\lambda_{\text{adv}}=1$, with no regularization as α=0 and β=0.

In contrast, our approach involves simultaneous training for H&E to the eight different stains, using a total of 9 encoders, 9 decoders and 9 discriminators. Training consists of 500 epochs at a fixed learning rate of $2\;\times \;10^{-4}$, followed by 500 decay epochs with a linearly decaying rate. The loss weights and regularization settings are identical to those used in the CycleGAN models.

The values presented in S1 Table represent the mean tile-wise (no overlap) MSE for each stain tested on the Crohn’s dataset (see Sect 2.2). These MSE values are computed for both approaches – our unified method and CycleGAN – and are reported in S1 Table.

#### 2.7.2. Impact of incorporating IHC loss functions and H&E regularization on stain synthesis quality.

In S2 Table, the training involved 9 encoders, 9 decoders, and 9 discriminators. The model underwent 500 epochs at a fixed learning rate of $2\;\times \;10^{-4}$, followed by 500 decay epochs with a linearly decaying rate. The impact of incorporating different loss functions and regularization was studied, specifically:

- ${ℒ}_{\text{H\&E}}$ (✓): This regularization was applied at the end of each iteration, where the cycle consistency losses ${ℒ}_{\text{cyc},i}$ from the 8 components of the Crohn dataset were summed and averaged. The loss weights were set as $\lambda_{\text{cyc}}=10$ and $\lambda_{\text{adv}}=1$, with α=0 and β=0.
- ${ℒ}_{\text{IHC}}$ (✓): For IHC-specific loss, $\lambda_{\text{cyc}}=10$ and $\lambda_{\text{adv}}=1$ were maintained and the values of *α* and *β* were calculated as detailed in Sect 2.3.1.
- Combined ${ℒ}_{\text{H\&E}}$ (✓) and ${ℒ}_{\text{IHC}}$ (✓): Both H&E regularization and IHC loss were applied in a similar way as described above, with values of *α* and *β* calculated according to the method described in Sect 2.3.1.

The values presented in S2 Table represent the MSE, PSNR, and SSIM computed at the WSI level. These metrics are calculated for WSIs reconstructed with overlap 0% and represent the mean values for all eight different stains of the Crohn dataset. More details on the validation protocol are provided in S1 Text.

#### 2.7.3. Comparison of our model’s performance across different magnifications.

In S3 Table, we evaluated performance in paired and unpaired settings using specific magnifications. This involved 9 encoders, 9 decoders, and 9 discriminators. The model underwent 500 epochs at a fixed learning rate of $2\;\times \;10^{-4}$, followed by 500 decay epochs with a linearly reducing rate. The magnifications tested were:

- x10 with an original tile size of 2048 x 2048 pixels, which corresponds to approximately 450.56×450.56μm,
- x20 with an original tile size of 1024 x 1024 pixels, approximately 225.28×225.28μm,
- x40 with an original tile size of 512 x 512 pixels, approximately 112.64×112.64μm.

All images are resized to 512x512 for training, following the configuration detailed in Sect 2.7.2. This configuration employs combined loss functions ${ℒ}_{\text{H\&E}}$ and ${ℒ}_{\text{IHC}}$, with parameters $\lambda_{\text{cyc}}=10$ and $\lambda_{\text{adv}}=1$. Performance metrics, listed in S3 Table, include MSE, PSNR, and SSIM. These metrics are computed on reconstructed WSIs with 0% overlap (mean values across all eight different stains of the Crohn dataset). It is important to note that training at a magnification of x40 and testing at x10 require resizing the synthetic x40 WSI to match the size of the x10 slide. After resizing, metrics are calculated to compare the ground truth slide at x10 with the resized slide. This procedure is also applicable to other magnifications. More details on the validation protocol between two WSIs are provided in S1 Text.

#### 2.7.4. Effects of various regularization techniques on unpaired virtual staining performance.

In S4 Table, we evaluated the performance under an unpaired setting using x10 magnification (original tile size of 2048x2048 pixels, corresponding to approximately 450.56×450.56μm), resized to 512x512. The first row details our approach using the configuration described in Sect 2.7.1. The second row uses the same configuration, combining the IHC loss functions with H&E regularization, as referenced in Sect 2.7.2. For subsequent rows, whenever a specific stain regularization is applied, the parameters $\lambda_{\text{cyc}}=10$, $\lambda_{\text{adv}}=1$, and values for *α* and *β* are computed according to the method described in Sect 2.3.1. In addition, ${ℒ}_{\text{idt}}=1$, ${ℒ}_{\text{lat}}=1$, or ${ℒ}_{\text{fwd}}=1$ can be applied, with detailed descriptions of each stain regularization found in Sect 2.3.2.

## Results

### 2.8. Study of unified vs. individual H&E encoders in multi virtual staining

In our study, our objective was to develop an enhanced technique for generating multiple virtual stains simultaneously. Existing methods, as discussed in Sect 2.1.1, are limited to producing at most three stains concurrently. Inspired to improve upon these limitations, we utilized style transfer techniques from frameworks such as ComboGAN [25], which can handle up to 14 different art styles. We adapt this approach to histopathological applications, incorporating a novel architecture with a dedicated H&E encoder, generator, and discriminator for the concurrent training of H&E to multiple *S* stains, as illustrated in Sect 2.3 - S1, S4 and S5 Figs.

Our results, detailed in S1 Table, demonstrate the advantages of using a unified H&E encoder for multi virtual staining. Synthetic stains from this encoder consistently surpassed those of separate encoders for each stain. We evaluated performance using the mean square error (MSE), comparing synthetic stains with authentic counterparts with a paired test set of H&E samples. This comparison revealed that our method significantly outperforms the CycleGAN approach.

The unified and CycleGAN methods were tested under identical conditions, including the same dataset, training duration, and architecture for encoders, generators, and discriminators. Our method not only offers significant gains in computational efficiency by employing a single encoder, decoder, and discriminator throughout the staining process, but also requires fewer trainable parameters than the CycleGAN approach (see S3 Fig). This streamlined architecture boosts computational efficiency and supports scalability, accommodating a broader range of output stains, and accelerating the training process.

In conclusion, the unified H&E encoder method excels in producing more accurate synthetic stains and achieving greater computational efficiency, making it a scalable and effective solution for large-scale histopathological studies.

This approach also enables the development of robust H&E representations for downstream tasks. We evaluated our pretrained H&E encoder on a HER2-positive vs. HER2-negative classification task from H&E patches. Training from our pretrained H&E encoder weights demonstrated faster convergence (20 versus 110 epochs) and superior performance (AUC 0.877 versus 0.840) compared to the model trained from scratch with random initialization. These results highlight the transferability of the learned H&E features, suggesting that the unified encoder captures generalizable tissue morphology and microenvironmental characteristics that are valuable for various applications beyond virtual staining. More details on the classification method are provided in S1 Text. A per-stain evaluation (see S1 Text.) further demonstrated that reconstruction fidelity depends on subcellular localization, with membrane-associated and pan-cellular stains (e.g. D2-40 and GIEMSA) exhibiting high PSNR and SSIM.

### 2.9. Annotation-free knowledge guided training and overall H&E regularization

To improve the trustworthiness and robustness of virtual staining techniques in histopathology, we developed a novel methodology that incorporates additional constraints into the training model by leveraging information from stained slides. Contrary to style transfer applications in art [23–25], where domains differ significantly, simplifying the discriminator’s task and placing greater emphasis on the generator for accurate image synthesis, functional staining challenges are rooted in common morphological features in all stains, with variations primarily in activation reactions targeting specific proteins. This introduces two main challenges: (i) the model may underestimate regions with activated stains, which are statistically less frequent than non-activated tissue areas, leading to errors in stain generation as the discriminator struggles to differentiate between true activated regions and false negatives generated by the model; (ii) as the number of output stains increases, the encoder may disproportionately prioritize certain stains, potentially skewing the learning process and impeding overall performance.

To address these challenges, our approach incorporates ${ℒ}_{\text{IHC}}$ loss functions that automatically recognize stain-specific properties (see S2 Fig), and adaptively modulate the loss functions to emphasize underrepresented activated regions (refer to Sect 2.3, S4 and S5 Figs), thereby minimizing errors and reducing hallucinations. Stain-activated regions are first identified and then used to spatially weight the loss functions, improving the focus on the relevant tissue areas.

Furthermore, we introduce an H&E regularization ${ℒ}_{\text{H\&E}}$ to maintain balanced attention across various stains, recalibrating the model to equally consider all stains by backpropagating the mean error of generated stains through the H&E components exclusively.

This strategy not only stabilizes the training process but also scales effectively, as demonstrated by the improved results in S2 Table. The combination of ${ℒ}_{\text{IHC}}$ with ${ℒ}_{\text{H\&E}}$ enhances the performance of the model in paired and unpaired settings, ensuring consistent focus across different stains and significantly increasing overall efficacy.

### 2.10. Context-importance in multi virtual staining quality and scalability

Due to the enormous size of WSIs, which typically measure around 10,000 x 10,000 pixels, current GPUs cannot process an entire slide at once during training. As a result, virtual staining techniques often utilize a sliding window tiling approach. This method involves dividing the slide into smaller, more manageable patches that are compatible with deep learning models and GPU capacities. The common dimensions for these patches are 128 x 128 pixels and 256 x 256 pixels [22,44]. Using this approach requires careful consideration of the optimal magnification level (x10, x20, and x40) for analysis. The choice of magnification impacts the training process in paired and unpaired learning scenarios. Using smaller patches increases the total number of patches per WSI, raising questions about inference time – specifically, the duration required to reconstruct a virtually stained slide. Addressing these technical issues is crucial not only for optimizing performance but also for understanding the practical implications related to inference time, a critical factor for pathologists.

In our empirical experiments, using a modular approach that does not require loading all model parts simultaneously, we were able to process 512 x 512 tiles while simultaneously outputting 8 stains plus H&E during training on a standard 16GB GPU. This setup provides at least four times more spatial resolution than those reported in [22,44], thus offering more flexibility and the ability to incorporate more context within each patch. To determine the optimal magnification level for virtual staining, we trained our model using various magnifications, each resized to a uniform dimension of 512 x 512 pixels for consistent image processing. The specific magnifications tested were x10 (original tile size of 2048 x 2048 pixels ≈ 450.56 x 450.56μm), x20 (original tile size of 1024 x 1024 pixels ≈ 225.28 x 225.28μm), and x40 (original tile size of 512 x 512 pixels ≈ 112.64 x 112.64μm). These experiments were carried out in paired and unpaired learning settings to evaluate the impact of magnification on model performance. As shown in S3 Table, in the paired setting, all magnifications yielded comparable results due to the direct correspondence between the H&E-stained WSI and other stained WSIs. Our experiments in unpaired settings revealed a valuable insight: lower magnifications, which provide broader contextual views, enhance the performances. This suggests that embracing more extensive contextual information is crucial for effective learning – where direct stain correspondences are lacking –, guiding future improvements in virtual staining techniques.

In the paired analysis, we initially utilized a 10x magnification, corresponding to a resolution of 512 x 512 pixels (approximately 0.88 μm per pixel). We further processed the original images by resizing them to 1024 x 1024 pixels (0.44 μm per pixel) to better evaluate the impact of pixel density on high-context paired training. To maximize the full capabilities of high-end GPUs, such as the NVIDIA A100 80GB, we also experimented with a maximum image size of 1400 x 1400 pixels (0.32 μm per pixel) during the training phase.

S5 Table illustrates the scalability of our modular approach, which can successfully process images with eight stains plus H&E up to the resolution of 1400 x 1400. The comparative results in S3 and S5 Tables emphasize that improving contextual information within images proves substantially more advantageous than increasing the density of pixels.

### 2.11. Regularization techniques impact on unpaired multi virtual staining quality

Most style transfer methods emphasize the importance of regularization in improving and stabilizing the training process. For example, regularization of identity mapping loss is critical in preserving the color of input paintings in artistic applications, as seen in the CycleGAN [17] framework. Similarly, forward loss regularization plays a crucial role in virtual staining by preserving morphological characteristics when translating from H&E staining to other types, as highlighted in the UMDST [44] model. Despite the variety of available regularization techniques, comprehensive ablation studies are lacking to evaluate their effectiveness in virtual staining.

In our research, presented in S4 Table, we conducted an extensive ablation study to evaluate the individual and combined effects of various regularization techniques on the quality of virtual stain synthesis. This study examines different combinations of synthesis loss functions and regularization methods to identify the most effective configurations. The metrics used in the study include MSE, PSNR, and SSIM, which gauge the error, quality, and visual similarity of the synthesized images, respectively.

Our results, as presented in S4 Table, offer a detailed analysis of how various combinations of loss functions—specifically identity loss ${ℒ}_{\text{idt}}$, latent loss ${ℒ}_{\text{lat}}$, and forward loss ${ℒ}_{\text{fwd}}$ (refer to Sect 2.3.2)—impact key performance metrics such as MSE, PSNR, and SSIM. Each row in the table represents a different combination of these loss functions, illustrating their respective effects on the evaluation metrics. This detailed evaluation provides essential information on the efficacy of each approach.

First, applying the forward loss ${ℒ}_{\text{fwd}}$ alone has demonstrated superior results compared to baselines that include or exclude the combination of ${ℒ}_{\text{IHC}}$ and ${ℒ}_{\text{H\&E}}$. The ${ℒ}_{\text{fwd}}$ is particularly effective as it assists the model in preserving the morphological features that are highlighted by the H&E staining, crucial for accurate virtual staining.

Secondly, the best performance is achieved when ${ℒ}_{\text{fwd}}$ is combined with ${ℒ}_{\text{idt}}$. This combination not only preserves the morphological integrity of the stains but also maintains the original features of the input images, thereby ensuring high fidelity in the virtual staining process. This suggests that integrating both forward and identity losses provides a robust method to enhance the quality and accuracy of the synthesized stains, making it particularly suitable for applications requiring high precision in unpaired virtual staining.

In contrast, the inclusion of latent loss ${ℒ}_{\text{lat}}$ in the combinations tested does not appear to contribute positively to the staining results. In fact, configurations incorporating ${ℒ}_{\text{lat}}$ consistently underperformed in all metrics compared to those without it. This observation suggests that latent loss may interfere with the preservation of crucial stain-specific characteristics, thus making it a less desirable option in the context of virtual staining, where accuracy and fidelity are paramount.

### 2.12. Mitigating stitching artifacts in WSI virtual staining

In our review of existing virtual staining approaches [6,11,12,19,21,22,44], both paired and unpaired, it is evident that most employ a sliding window tiling approach during model training, as discussed in Sects 2.1.1 and 2.10. This training method inevitably leads to challenges in reconstructing WSIs from the resultant patches. In particular, it can cause visible artifacts, such as sudden color changes at tile borders and errors near these boundaries, as demonstrated in S7B Fig (0% overlap in both settings, marked with red arrows). These artifacts not only undermine the trust of pathologists in the tools but can also increase cognitive load and error rates during slide examination. This issue is pervasive across all tile-based virtual staining methods, underscoring the necessity for a universal solution.

To mitigate these problems, we developed a post-processing technique tailored for WSIs in the context of virtual staining. Our observations indicate that the models are context-sensitive, performing with high accuracy at the tile’s center and less so near the edges. Using this insight, our approach involves stitching tiles with an intentional overlap that prioritizes the central regions of the tiles using a Hamming [39,40] window (refer to Sect 2.5), effectively enhancing performance without additional training. This method, depicted in S7 Fig, significantly improves all evaluated metrics in both paired and unpaired settings and results in higher perceived image quality relative to the ground-truth. To further refine our approach, it is important to note that while our post-processing technique does introduce a slight increase in processing time, it offers a significant benefit in terms of performance-to-time ratio. An optimal overlap of 60%, as illustrated in the figures, provides the best balance between performance enhancement and execution time (>1min per 8 stains).This post-hoc processing strategy not only effectively addresses stitch artifacts, but also enhances the overall utility of virtual staining technologies in clinical settings. By maintaining a streamlined workflow, it facilitates the routine use of these technologies in a fast-paced clinical environment, potentially broadening their adoption and building trust among pathologists. This adjustment ensures the high quality of the virtual WSI stain generated (refer to S6 and S13 Figs) while offering spatial context, keeping processing time manageable, thus aligning with the needs and dynamics of modern anatomopathological practice.

### 2.13. Trust in virtual stains through self-inspection–anomaly detection

A significant barrier to integrating generative models, particularly in healthcare, is the absence of a confidence score with the generated output. This limitation raises critical questions: How can we detect problems in the input H&E data? What is the model’s confidence in its virtual stains? How can we assess the quality of synthetic stains and identify errors that might influence a pathologist’s decision to rely on virtual stains or request traditional chemical stains for confirmation?

Such concerns are paramount in high-stakes decision-making processes. There is a pressing need for interpretable methods to ensure that these powerful generative approaches are not sidelined because of a lack of trustworthiness. In this study, we leverage knowledge-guided training not only to improve control over the learning process-which has demonstrated improvements in performance (refer to Sect 2.9)—but also to provide pathologists with an interpretable narrative. This approach considers stain masks, shifting the model’s focus to medically relevant features, thus providing a clearer explanation than a fully black-box model.

In addition, we use the learned knowledge of the discriminator in the training phase, an element that is often discarded after training, which models the authenticity of images. This unique application allows the inspection of data quality and its deviation from the learned distribution.

To demonstrate the utility of this approach, we processed H&E tiles and examined global degradation that could arise from incorrect stain concentration or scanner configurations, as illustrated in S9 Fig. The discriminator effectively detects the domain shift in these degraded images, aligning with an anomaly detection framework. It flags deviations from the learned distribution in red, as shown in S9 Fig. These results support the hypothesis that H&E tiles presenting new defects can be detected using the model discriminator.

An extensive evaluation was conducted on 2,022 authentic WSIs. This H&E only dataset, which cannot be publicly released due to privacy constraints, consists of slides from pediatric Crohn’s disease patients followed at Robert Debré Hospital (Paris, France). Slides (5 *μ*m thick) were stained with H&E, digitized using a Hamamatsu slide scanner, and semi-anonymized prior to analysis. The dataset comprises 47,984 tiles (512 × 512 H&E tiles).

Given that anomalies can be local or global, we employed the standard deviation of the discriminator’s confidence map as an indicator, as depicted in S8 Fig. This analysis not only confirms that the discriminator can detect outliers (e.g., artifact tiles, mostly background tiles) but also facilitates the empirical determination of a confidence interval (e.g., 3.11% ≤ acceptable ≤ 14.86%). This method introduces an effective filter to avoid feeding the multi virtual staining approach with unsound H&E images (garbage in garbage out), thus, reduces the potential error rate in synthetic stains, enhancing reliability and trust in generated outputs.

In addition, we employed the discriminator’s confidence maps on the outputted virtual stains to generate pixel-wise confidence scores. These scores empower pathologists by highlighting regions where the virtual staining deviates from the expected representation of a stain. This feature acts as a secondary filter in the output stage of our pipeline, visually represented through heatmaps as depicted in S10 Fig. The Figure contrasts the discriminator’s responses to identical tissue sections—one from an authentically stained WSI and the other from a virtually stained WSI with a staining error. In particular, the discriminator indicates red staining discrepancies, aligning with the actual differences computed between the authentic and virtual images. This methodology demonstrates the approach’s ability to provide pathologists with a reliable confidence score, offering additional context to determine the significance of the region in question for specific use cases. It also assesses whether it is necessary to perform a chemical stain to confirm findings, thus eliminating any uncertainties. By clearly indicating areas of uncertainty in the output, this tool builds greater trust in virtual staining technologies, reassuring users of its reliability, and enhancing overall confidence in the outputs provided.

This study highlights the quality check capability of integrating discriminator confidence maps into the workflow of digital and virtual staining in pathology (refer to S1 Fig). Our approach’s ability to identify discrepancies and artifacts at both input and output stages ensures that only high-quality, reliable data are utilized and generated, addressing the critical issue of "garbage in, garbage out" in medical imaging. In the future, the adoption of such advanced tools promises to refine the precision of digital pathology, potentially leading to more personalized and timely therapeutic interventions.

### 2.14. Cloud-based digital pathology for enhanced efficiency and usability–proof-of-concept

Configuring the software and hardware required for complex generative models can be time consuming and resource intensive, demanding specific technical skills that may not be readily available in the busy environments typical of pathology laboratories. An anywhere accessible system, preferably through a browser, could significantly enhance time efficiency and work comfort. In this study, our goal is to provide a holistic approach to managing multi virtual staining. Therefore, we have chosen to use Cytomine [35], an open-source platform, as a proof of concept to deploy our multi virtual staining technique (discussed in Sect 2.1.3). This choice allows us to bridge the gap between cutting-edge DL models and their day-to-day application, offering replicable guidelines for utilizing open-source, cloud-based platforms.

To achieve this, we deployed the platform and containerized our multi virtual staining implementation before integrating it into the cloud-based platform. This approach enables pathologists to easily execute complex algorithms directly through their web browser, as illustrated in S11 Fig. This streamlined integration simplifies the use of advanced DL models in routine pathological analysis, enhancing the accessibility and practicality of digital histopathological tools.

### 2.15. Paired H&E-multi-stains dataset in the context of pediatric Crohn’s disease at diagnosis

As detailed in Sect 2.1.2, one primary challenge in multi-stain data analysis is the scarcity of publicly available datasets. For example, the dataset of de Haan et al. (2021) was not shared [6]. Furthermore, the availability of high-quality paired data is limited; typically, datasets such as AHNIR [45] are compiled from adjacent slides, resulting in imperfectly matched samples. Specifically, the AHNIR kidney dataset contains only a limited set of slides, with five slides for each stain type: H&E, PAS, PASM, and MAS.

This issue is also prevalent in other studies as well; for example, MVFStain [22] used only a fraction of the AHNIR dataset for lung lesions, employing one WSI for training and another for testing. Similar methodologies are applied to datasets related to lung lobes and breast tissues, using two WSIs for training and one for testing to maintain methodological consistency.

These challenges not only hinder the public availability of such data, but also limit the diversity and quality of the datasets. With samples derived from adjacent slides, significant pairing challenges arise, complicating the objective evaluation of computational methods. This necessitates the use of elastic registration (e.g. VALIS [46]), which is error-prone due to variations in tissue characteristics.

To overcome these limitations, we propose the introduction of a new dataset that provides paired H&E to eight different stains, focusing on pediatric Crohn’s disease. This dataset aims to catalyze further research in computational pathology by including 30 H&E WSIs and 30 stained WSIs in eight stains (AE1AE3, CD117, CD15, CD163, CD3, CD8, D240, and GIEMSA), culminating in a total of 480 WSIs. Each sample consists of perfectly matched data from identical tissue sections, as depicted in S12 Fig. This comprehensive collection of 480 WSIs aims to drive advances in computational pathology. By providing high-quality, diverse data, we anticipate setting a new benchmark for methodologies not only in virtual staining but also in segmentation, detection, and other computational histopathology applications.

## Discussion

Our investigation of the current state of the art in computational pathology has revealed critical challenges, notably the opaque nature of deep learning technologies and a shortage of high-quality public data. These issues significantly hinder the integration of advanced computational tools into routine clinical practice. To address these challenges, our study proposes a holistic approach focused on enhancing performance, trustworthiness, scalability, and data quality and quantity. This approach ensures that complex systems are accessible through secure cloud-based platforms, which is crucial to their successful integration into the field.

The methodology we developed significantly contributes to computational pathology by improving scalability through compressive regularization and knowledge-guided methodologies during both the training and inference phases. Trust is further enhanced by incorporating discriminators for input quality control and output confidence scoring. The practical implementation of our model in an open-source, cloud-based deployment for virtual staining demonstrates promising potential for real-world applications.

In advancing the field’s understanding of virtual staining, we released a dataset of 480 whole slide images. This not only sets a new standard for quantitative evaluation in computational pathology but also supports diverse applications such as segmentation and detection. By making these resources available, we encourage the scientific community to engage in more reproducible research using this dataset.

Looking ahead, expanding our dataset to include a wider range of pathological conditions beyond pediatric Crohn’s disease will enhance the generalization of our model. In a stain-level analysis of reconstruction accuracy, the cytoplasmic marker exhibited the greatest discrepancy between ground truth and virtual staining, a finding that likely reflects the challenge of faithfully reproducing its fine filamentous architecture. We also note that our current panel lacks a purely nuclear immunomarker, which prevents the evaluation of model performance in exclusive nuclear localization. Incorporation of dedicated nuclear targets will be essential in future work to ensure robust virtual staining across all subcellular compartments.

In conclusion, our research introduces significant enhancements to computational pathology by integrating a unified H&E encoder, adapted loss functions, regularization techniques, and context-driven learning within a cloud-based framework. These advances not only meet, but exceed current standards of quality and trustworthiness in stain transformations, paving the way for a more reliable, accessible, and effective future in computational pathology, ultimately contributing to better clinical outcomes.

## Supporting information

S1 Fig Visual-XAI-enhanced trustworthy virtual staining approach. End-to-end virtual staining approach that generates synthetic IHC stains using a single H&E encoder and multiple stain decoders. The Quality Check (QC) protocol based on self-inspection characteristics uses trained discriminators to consolidate trust in the synthetic stains generated, ensuring the alignment of the new H&E slides with the trained distribution and validating the quality of the generated stained slides. Integration of cloud-based computing enhances accessibility and adoption by enabling pathologists to efficiently process large datasets from anywhere, while end-to-end system’s algorithms are handled in a back-end containerized environment.
(TIFF)

S2 Fig Automated Extraction of IHC-Activated Regions from Stained Tiles. For each instance, such as (from the top) CD8, CD117, CD163, the extraction process is visualized in a three-column format. The left column displays the original RGB stained tile (*X*_*i*_); the middle column depicts the conversion of the tile to the HSV color space, capturing the unique chromatic signature from antibody-tissue reactions; and the right column showcases the resulting binary mask (*M*_*i*_) highlighted in yellow.
(TIFF)

S3 Fig Comparison of H&E staining-based methodologies for virtual stain generation in computational histopathology during production phase. Panel **A** illustrates the proposed unified H&E encoder approach, adapting the ComboGAN [25] approach to virtual staining, employing a single encoder and multiple decoders to generate various synthetic stains, thereby optimizing computational efficiency and scalability (to maintain focus on comparative methodology details on XAI capabilities are presented in S1 Fig). Panel **B** depicts the traditional CycleGAN-like methodologies [16,17], which use multiple separate encoders and decoders for each stain, increasing the complexity of the model and computational demand. Panel **C** showcases the StarGAN-like approaches [22–24,44], using a style encoder and a single generator for multiple stains. While this architecture simplifies the model, it requires substantial computational resources and does not scale effectively, particularly as the number of stains increases (more stains, bigger generator), and still necessitates loading the large generator even for a subset of stains, leading to inefficiencies. The unified H&E approach in panel **A** represents a significant advance by reducing the need for multiple models and facilitating faster and more resource-efficient processing. This model is able to produce only the required stains, loading minimal model components into memory, reducing hardware requirements and computational costs in cloud-based deployments.
(TIFF)

S4 FigComprehensive representation of the training process for paired stain synthesis and computation of loss functions H&E ↔ stain *i.***A.** Details the first training cycle, starting with a paired real H&E image $X_{H\&E}$ and generating a corresponding stain *i* image ${\hat{Y}}_{i}$, followed by the reconstruction of the original H&E image ${\hat{X}}_{H\&E}$ to facilitate computation of the loss function components detailed in Sect 2.3. **B.** Maps the second training cycle, beginning with a paired real stain *i* image *X*_*i*_, producing a corresponding H&E image ${\hat{Y}}_{H\&E}$, and concluding with the reconstructed stain *i* image ${\hat{X}}_{i}$, using the staining mask *M*_*i*_ (${\bar{M}}_{i}$ corresponds to the complementary mask of *M*_*i*_) to compute various elements of the loss function detailed in Sect 2.3. Each panel illustrates the modifications of the model aimed at enhancing the precision and consistency of stain synthesis and discrimination in paired training scenarios.(TIFF)

S5 FigDetailed representation of the scalable training process for unpaired stain synthesis and computation of loss functions H&E ↔ stain *i.***A.** Illustrates the first training cycle, beginning with a real H&E image $X_{H\&E}$, generating a synthetic stain *i* image ${\hat{Y}}_{i}$, and closing with the reconstructed H&E image ${\hat{X}}_{H\&E}$ to enable computation of the loss function components. **B.** Demonstrates the second training cycle, starting with a real stain image *i X*_*i*_, producing a synthetic H&E image ${\hat{Y}}_{H\&E}$, and concluding with the reconstructed stain *i* image ${\hat{X}}_{i}$, incorporating the stain mask *M*_*i*_ (${\bar{M}}_{i}$ corresponds to the complementary mask of *M*_*i*_) to compute various elements of the loss function (refer to Sect 2.3). Each panel highlights different aspects of the model’s adaptations and refinements, targeting and enhancing underrepresented activated regions to ensure more accurate and consistent stain synthesis and discrimination.(TIFF)

S6 FigResults of multi virtual staining in the context of Crohn’s diseaseThis Figure shows the high resolution WSIs of various synthetic stains achieved using loss functions ${ℒ}_{\text{IHC}}$ and ${ℒ}_{\text{H\&E}}$ in a non-paired setting.(TIFF)

S7 Fig Illustration of the effects of post-processing on stitching artifacts and performance metrics in virtual staining. **(a)** Depicts the improved results using different overlap approaches with a Hamming window, emphasizing the enhanced image quality and reduced artifacts, with the optimal performance-time execution ratio achieved in overlap. 60%. **(b)** Shows typical stitching artifacts at the tile borders with overlaps 0%, 30% and 60%, marked by red arrows, demonstrating sudden color changes and errors near the boundaries. This Figure highlights the comparison across performance metrics (MSE, PSNR, SSIM) in both paired and unpaired settings, showcasing the effectiveness of the post-processing strategy in enhancing overall quality and facilitating the adoption of virtual staining technologies in clinical environments. For reproducibility details, refer to Sect 2.5.
(TIFF)

S8 Fig Discriminator confidence mapping for H&E tile authenticity evaluation (anomaly detection). This Figure evaluates the authenticity of 47984 H&E-stained tiles from 2022 authentic WSIs (H&E stained during 20 years of time interval with different scanners) using discriminator confidence maps. The standard deviation of the map is used to assess the authenticity of each tile. The histogram provides pathologists with an empirical tool to determine the acceptable H&E range (e.g., 3.11% to 14.86%), identifying tiles within this range as highly authentic. Tiles outside this range are flagged as outliers, typically due to being background or significantly degraded, indicated by unusually high or consistently low deviations on the confidence maps. These results highlight the discriminator’s ability to identify and quantify tile authenticity, serving as an essential tool for pathologists to exclude unreliable artifacts during the H&E staining and scanning processes. This approach effectively prevents the introduction of substandard images into the multi virtual staining pipeline, thereby reducing the potential error rate in synthetic stains and enhancing the reliability and trustworthiness of generated outputs. For reproducibility details, refer to Sect 2.4.
(TIFF)

S9 Fig Comparison of original and degraded H&E images with corresponding H&E discriminator’s confidence maps. Panels **A**, **B**, and **C** demonstrate the analysis of H&E-stained tiles. In each panel, the top row displays the original H&E tile alongside five degraded versions of the same tile, while the bottom row presents the associated discriminator’s confidence maps. These maps highlight areas of perceptual inconsistency marked in red. Panel **A** illustrates global degradation potentially caused by issues in chemical staining or scanning errors, such as incorrect staining concentration or scanner configuration problems, and the model successfully identifies such global defects. Panel **B** shows local contamination possibly due to chemical staining errors or physical artifacts on the scanner, the model pinpointing the locations of the contamination. Panel **C** depicts artifacts resembling water droplets that can adhere to slides during preparation, potentially causing analysis errors; here, the model indicates the positions of these droplet-like artifacts, thus drawing expert attention to the affected regions. For reproducibility details, refer to Sect 2.4.
(TIFF)

S10 Fig Visualization of discriminator confidence in virtual stain analysis. This figure illustrates the effectiveness of discriminator confidence maps in evaluating virtual and authentic stained WSIs. Two tissue sections are shown: one authentically stained and the other virtually stained with an identifiable error. The discriminator response is visualized through heatmaps, where areas of discrepancy are highlighted in red. These highlighted regions correspond to significant deviations from the expected stain appearance, providing pathologists with a pixel-wise confidence score. This visualization helps to determine the need for additional confirmatory chemical staining and to identify critical areas for detailed examination. By quantifying and displaying errors, this tool reinforces the reliability of virtual staining technologies and supports pathologists in making more informed decisions. For reproducibility details, refer to Sect 2.4.
(TIFF)

S11 Fig Toward effortless digital histopathology through cloud-based multi virtual staining: Proof-of-concept. **A.1.** displays a user interface for selecting the desired H&E WSI and setting the parameters for inference. **A.2.** illustrates the panel that tracks the progress of the multi virtual staining process (slurm job). **B.** presents synchronized views of a series of virtually stained slides alongside the original H&E slide (upper left). This Figure demonstrates our dockerized multi virtual staining implementation on the open source Cytomine platform [35] as a use case. Computations are performed on a back-end server (via slurm), with the user only required to upload the H&E slide and initiate the algorithm through the browser. The results are then displayed in a synchronized view, significantly minimizing user effort. For reproducibility details, refer to Sect 2.6.
(TIFF)

S12 Fig Visualization of the samples of the multi-stain pediatric Crohn’s disease dataset, showcasing paired H&E for different stain types. This Figure illustrates the perfect pairing of WSIs from identical tissue sections, which is central to the utility of the dataset in computational pathology research.
(TIFF)

S13 Fig Results of multi virtual staining on kidney slide N^°^5 from the AHNIR dataset. Showing the high-quality synthetic stains generated using our method.
(TIFF)

S14 Fig Evaluation protocol for the performance of virtual staining. Workflow diagram illustrating the validation process for virtual staining techniques. The process begins with an H&E stained whole slide image (H&E WSI), from which the foreground is extracted. This image undergoes virtual staining to produce the Stain WSI, which is then compared to the chemically stained ground-truth WSI (GT stain WSI). Evaluation metrics include PSNR and SSIM to assess overall image quality and MSE to evaluate pixel-wise accuracy, indicating the effectiveness of the staining simulation.
(TIFF)

S15 Fig Software for poll results and feedback collection of pathologist ratings on staining quality We show the original H&E image at the top, followed by a set of virtual stains in different conditions, including the ground truth randomly shown. The pathologist was asked to rate each image based on the clarity and preservation of morphological details. 1 "worst" 5 "best" with feedback.
(TIFF)

S16 Fig Morphological detail comparison in H&E stained images. This Figure shows a closer view of the morphological features in the original H&E stain (left) versus virtual ground truth, paired and unpaired stains. The comparison highlights the impact of the water-like blur in chemical stains and its reduction in virtual stains, aiding in the qualitative evaluation by pathologists.
(TIFF)

S17 Fig Comparison of test loss during training for HER2-positive patches classification from scratch and from pretrained H&E encoder. This Figure shows binary cross entropy in the test set during training of a binary classifier from our pretrained H&E encoder and a xavier initialization. The results highlight a faster convergence and better performance when using the pretrained H&E encoder, suggesting the potential of our methods for capturing and transferring protein expression-related features.
(TIFF)

S1 Table Performances and efficiency of the multi virtual staining using unified H&E encoder. This table compares the mean square error (MSE) metrics (mean±std) of synthetic stain generation (unpaired setting) using our unified H&E encoder versus traditional distinct H&E encoders per stain (CycleGAN). The results highlight our method’s superior accuracy and computational efficiency, featuring a significantly reduced number of trainable parameters, thus demonstrating its potential for scalable clinical-effective histopathological applications. For reproducibility details, refer to Sect 2.7.1.
(TIFF)

S2 TableImpact of incorporating ${ℒ}_{IHC}$ loss Functions and ${ℒ}_{H\&E}$ regularization on stain synthesis quality.Comparative results displayed for paired and unpaired staining settings, quantified by MSE, peak signal-to-noise ratio (PSNR) and structural similarity index (SSIM). For reproducibility details, refer to Sect 2.7.2.(TIFF)

S3 TableAnalysis of the performance of our model at different magnifications.This table presents the performance of our modular training approach at different magnifications (x10, x20, x40), where tiles extracted at each magnification were resized to 512 x 512 pixels to ensure a consistent image size for analysis. The models were trained using ${ℒ}_{\text{IHC}}$ and ${ℒ}_{\text{H\&E}}$ loss functions. In the paired setting, no particular magnification preference was observed, indicating uniformity in performance. In contrast, in the unpaired setting, lower magnifications, which provide more contextual information, demonstrated a significant advantage, underscoring the importance of context for effective learning in scenarios lacking direct correspondence between stain types. For reproducibility details, refer to Sect 2.7.3.(TIFF)

S4 TableEffects of various regularization techniques on the performance of virtual unpaired staining.This table displays an ablation study of different combinations of synthesis loss functions (${ℒ}_{\text{IHC}}$, ${ℒ}_{\text{H\&E}}$ detailed in Sects 2.3.1 and 2.3.2) and regularization methods (${ℒ}_{\text{idt}}$, ${ℒ}_{\text{lat}}$and ${ℒ}_{\text{fwd}}$ detailed in Sect 2.3.2) on the performance metrics MSE, PSNR and SSIM. Each row represents a specific configuration of loss functions, illustrating their impact on the accuracy and quality of virtual staining results. For reproducibility details, refer to Sect 2.7.4.(TIFF)

S5 Table Scalability of our multi virtual staining approach at various training resolutions in a paired setting. This table displays the results of training our virtual staining model on images with eight stains plus H&E at different resolutions. The results demonstrate consistent performance across various densities of pixels. The data highlight our approach’s effective use of advanced GPU resources, emphasizing the scalability of our methodology.
(TIFF)

S6 Table Comparative analysis of CSS for different staining methods across different models This table presents the CSS metrics for various computational methods when applied to human kidney tissue slides stained with H&E, MAS, PAS, and PASM. The performance of each method is evaluated in terms of overall CSS, tile output, WSI-compliant output and evaluation, XAI capabilities, and scalability. The results highlight our method’s superior ability to address the challenges of multi virtual staining, with higher CSS values signifying enhanced preservation of structural similarity across different stains.
(TIFF)

S7 Table Quantitative per-stain evaluation of virtual staining fidelity. This table reports, for each of the eight stainings, the mean squared error (MSE), computed on the masked tissue region, peak signal-to-noise ratio in decibels (PSNR), and structural similarity index (SSIM) computed over the entire tissue region of the WSI.
(TIFF)

S1 Text Supplementary materials include the generalization of the approach to different stain types, the validation protocol used for virtual staining, and additional information about the test IDs from the Crohn’s dataset for reproduction purposes. (PDF)
