# Supplementary figures and images for: Scalable, trustworthy generative model for virtual multi-staining from H&E whole slide images
Source: PLoS Comput Biol. 2025 Oct 21;21(10):e1013516. doi: 10.1371/journal.pcbi.1013516 (PMC12578341; doi:10.1371/journal.pcbi.1013516)

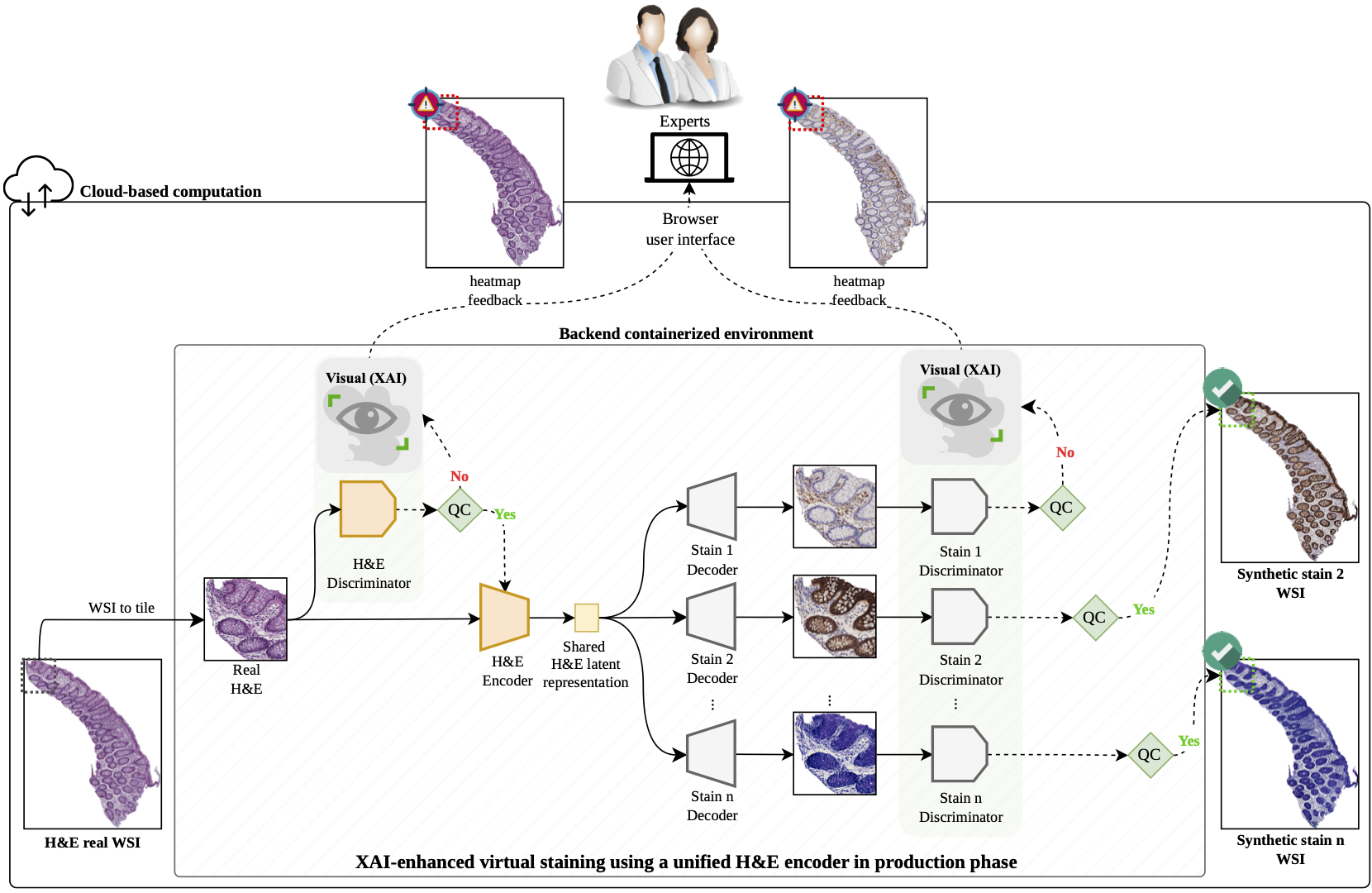

Supplement: S1 Fig — End-to-end virtual staining approach that generates synthetic IHC stains using a single H&E encoder and multiple stain decoders. The Quality Check (QC) protocol based on self-inspection characteristics uses trained discriminators to consolidate trust in the synthetic stains generated, ensuring the alignment of the new H&E slides with the trained distribution and validating the quality of the generated stained slides. Integration of cloud-based computing enhances accessibility and adoption by enabling pathologists to efficiently process large datasets from anywhere, while end-to-end system’s algorithms are handled in a back-end containerized environment. (TIFF) [file pcbi.1013516.s001.tiff]

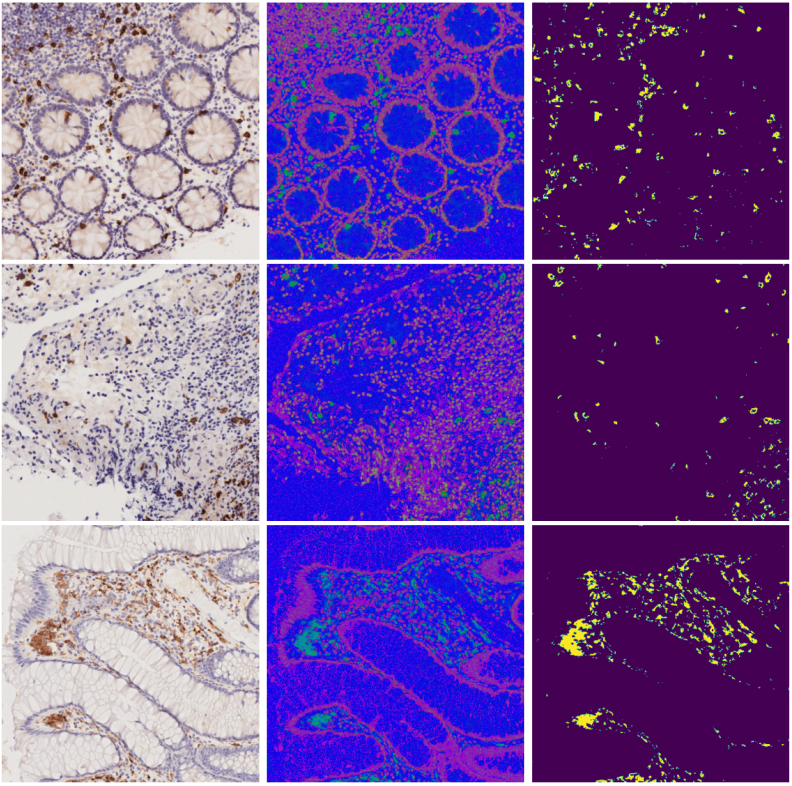

Supplement: S2 Fig — For each instance, such as (from the top) CD8, CD117, CD163, the extraction process is visualized in a three-column format. The left column displays the original RGB stained tile (Xi); the middle column depicts the conversion of the tile to the HSV color space, capturing the unique chromatic signature from antibody-tissue reactions; and the right column showcases the resulting binary mask (Mi) highlighted in yellow. (TIFF) [file pcbi.1013516.s002.tiff]

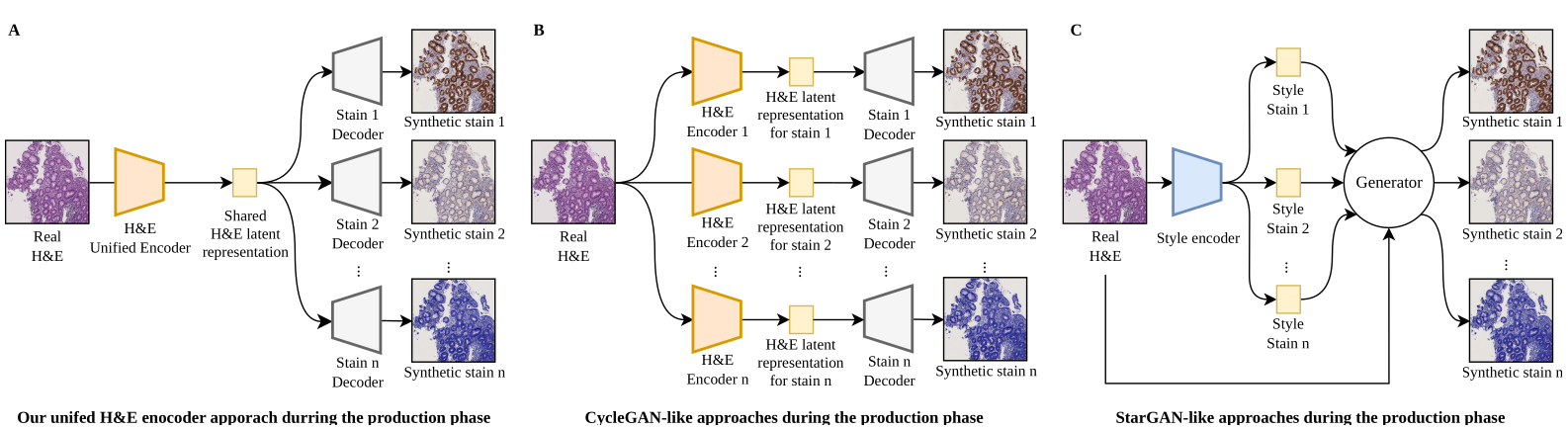

Supplement: S3 Fig — Panel A illustrates the proposed unified H&E encoder approach, adapting the ComboGAN [25] approach to virtual staining, employing a single encoder and multiple decoders to generate various synthetic stains, thereby optimizing computational efficiency and scalability (to maintain focus on comparative methodology details on XAI capabilities are presented in S1 Fig). Panel B depicts the traditional CycleGAN-like methodologies [16,17], which use multiple separate encoders and decoders for each stain, increasing the complexity of the model and computational demand. Panel C showcases the StarGAN-like approaches [22–24,44], using a style encoder and a single generator for multiple stains. While this architecture simplifies the model, it requires substantial computational resources and does not scale effectively, particularly as the number of stains increases (more stains, bigger generator), and still necessitates loading the large generator even for a subset of stains, leading to inefficiencies. The unified H&E approach in panel A represents a significant advance by reducing the need for multiple models and facilitating faster and more resource-efficient processing. This model is able to produce only the required stains, loading minimal model components into memory, reducing hardware requirements and computational costs in cloud-based deployments. (TIFF) [file pcbi.1013516.s003.tiff]

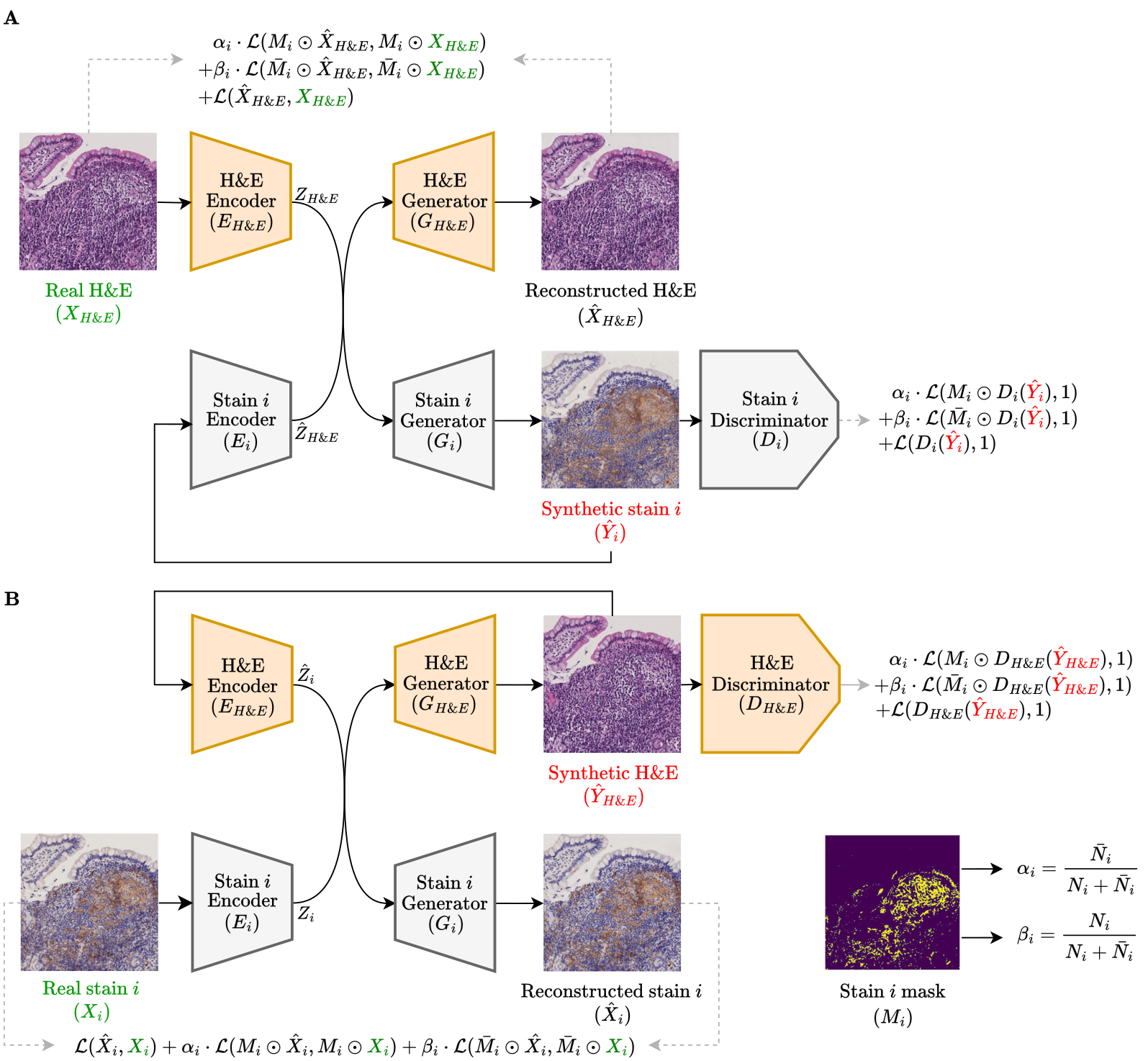

Supplement: S4 Fig — A. Details the first training cycle, starting with a paired real H&E image XH&E and generating a corresponding stain i image Y^i, followed by the reconstruction of the original H&E image X^H&E to facilitate computation of the loss function components detailed in Sect 2.3. B. Maps the second training cycle, beginning with a paired real stain i image Xi, producing a corresponding H&E image Y^H&E, and concluding with the reconstructed stain i image X^i, using the staining mask Mi (M¯i corresponds to the complementary mask of Mi) to compute various elements of the loss function detailed in Sect 2.3. Each panel illustrates the modifications of the model aimed at enhancing the precision and consistency of stain synthesis and discrimination in paired training scenarios. (TIFF) [file pcbi.1013516.s004.tiff]

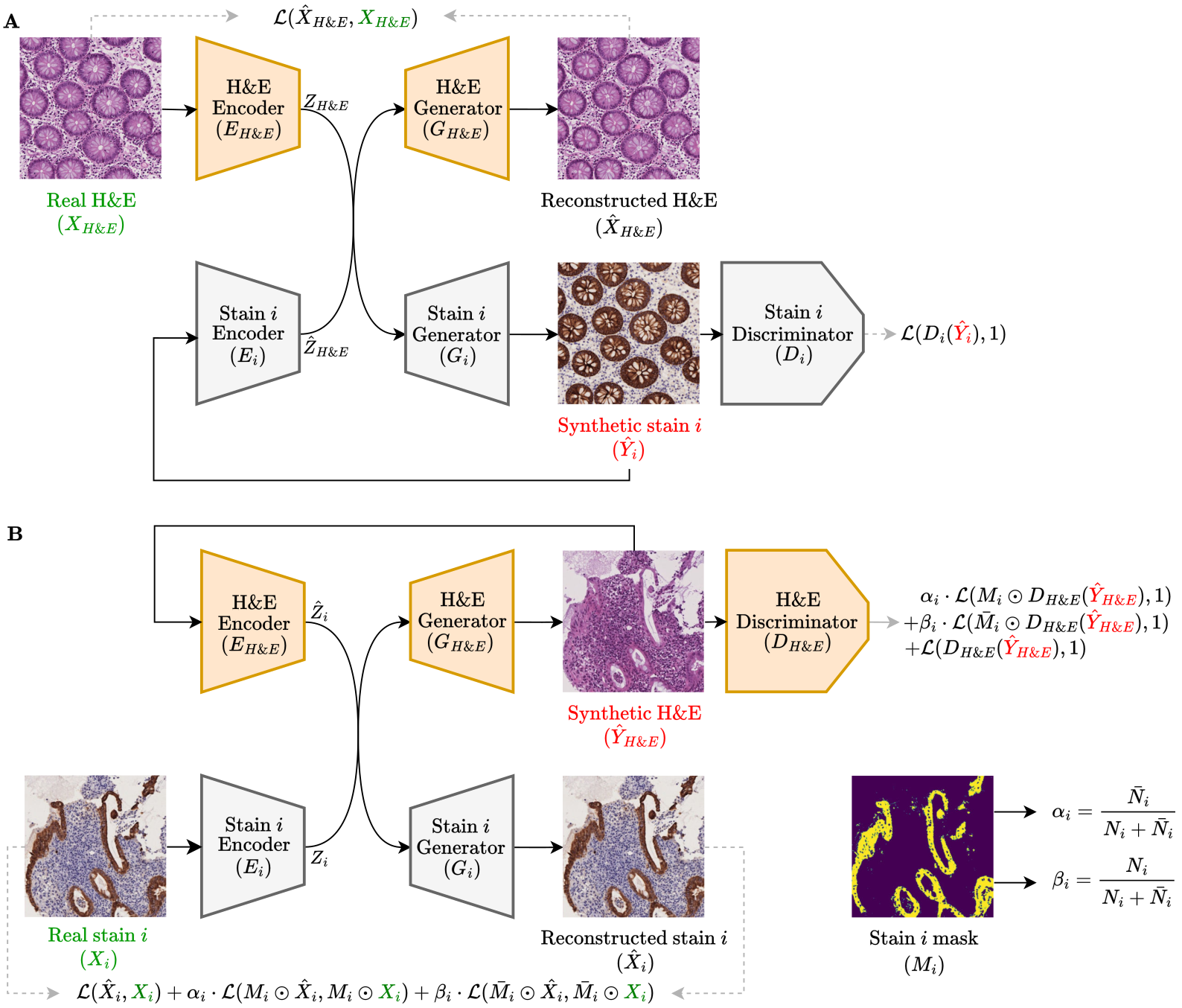

Supplement: S5 Fig — A. Illustrates the first training cycle, beginning with a real H&E image XH&E, generating a synthetic stain i image Y^i, and closing with the reconstructed H&E image X^H&E to enable computation of the loss function components. B. Demonstrates the second training cycle, starting with a real stain image i Xi, producing a synthetic H&E image Y^H&E, and concluding with the reconstructed stain i image X^i, incorporating the stain mask Mi (M¯i corresponds to the complementary mask of Mi) to compute various elements of the loss function (refer to Sect 2.3). Each panel highlights different aspects of the model’s adaptations and refinements, targeting and enhancing underrepresented activated regions to ensure more accurate and consistent stain synthesis and discrimination. (TIFF) [file pcbi.1013516.s005.tiff]

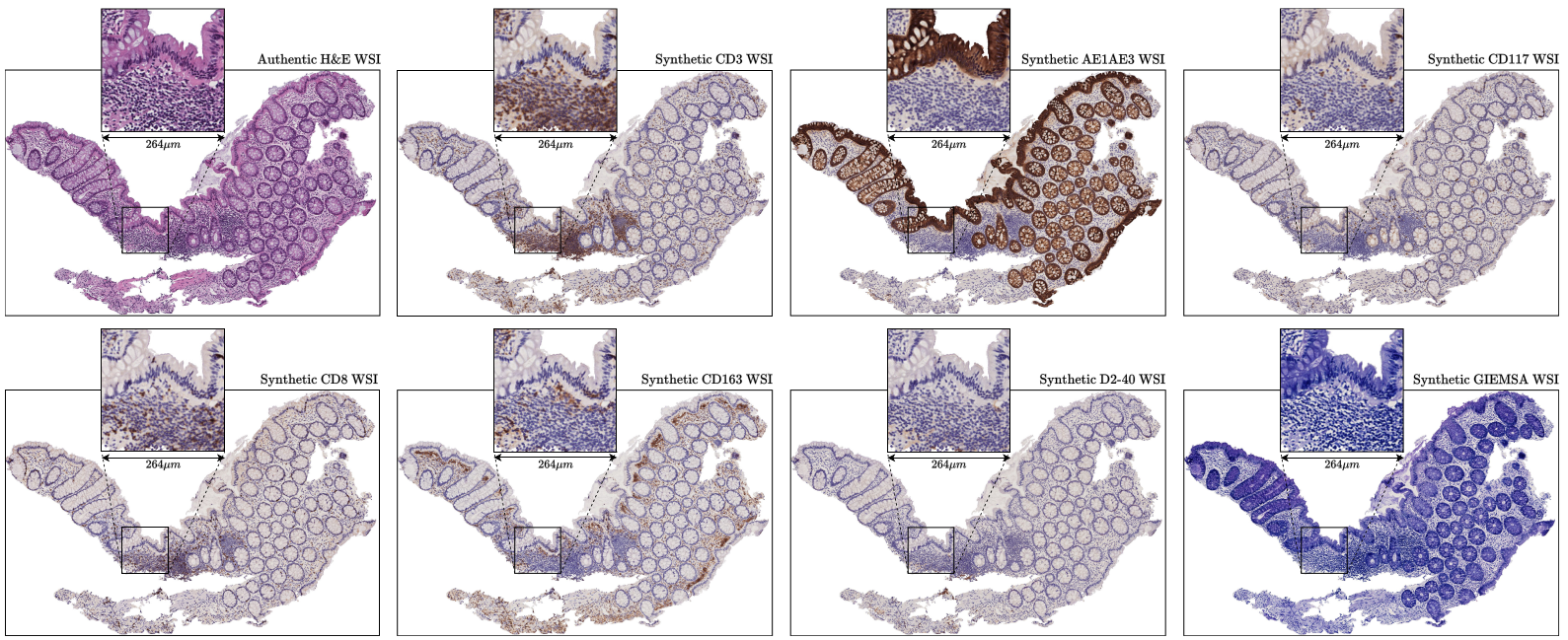

Supplement: S6 Fig — This Figure shows the high resolution WSIs of various synthetic stains achieved using loss functions ℒIHC and ℒH&E in a non-paired setting. (TIFF) [file pcbi.1013516.s006.tiff]

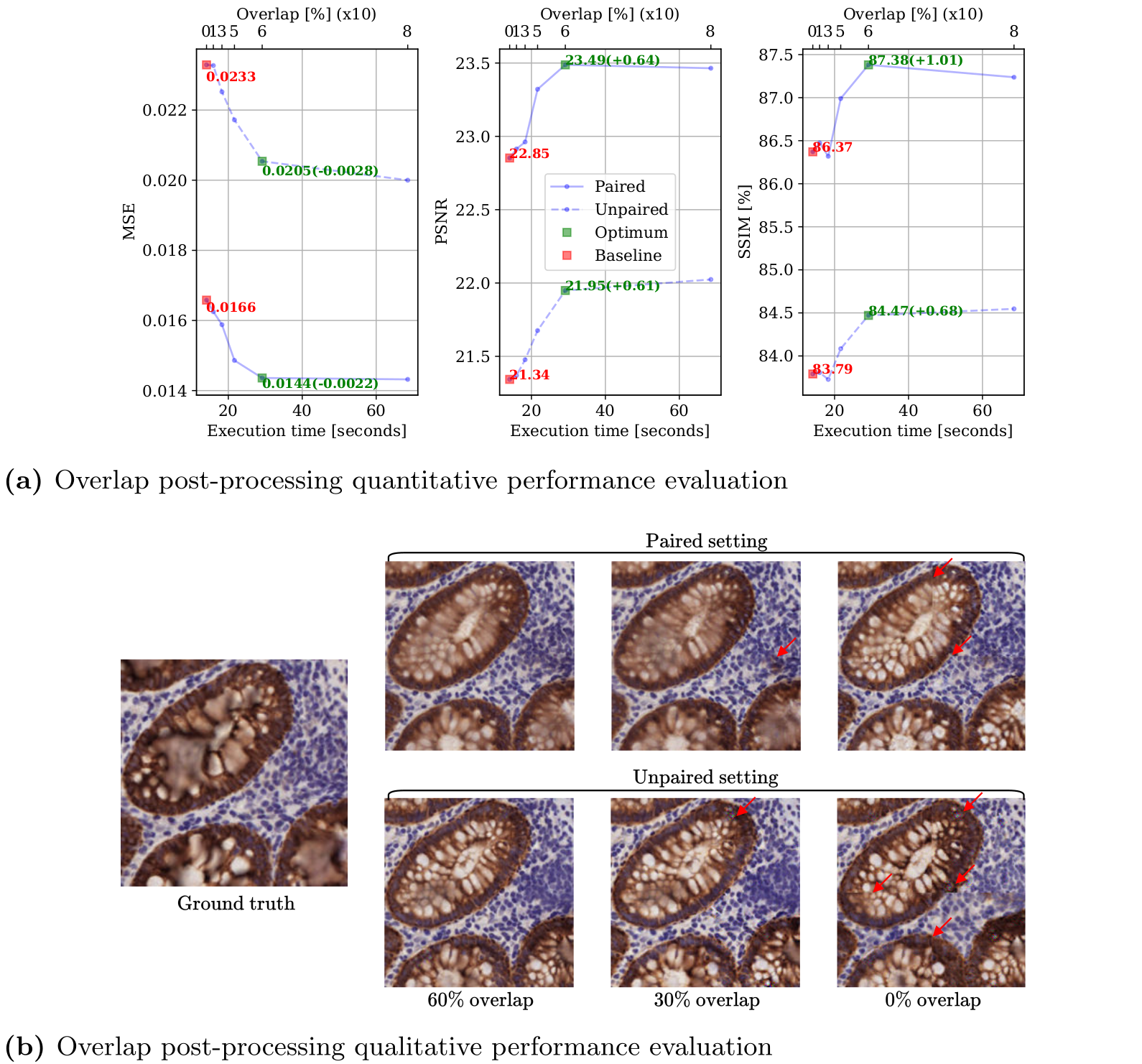

Supplement: S7 Fig — (a) Depicts the improved results using different overlap approaches with a Hamming window, emphasizing the enhanced image quality and reduced artifacts, with the optimal performance-time execution ratio achieved in overlap. 60%. (b) Shows typical stitching artifacts at the tile borders with overlaps 0%, 30% and 60%, marked by red arrows, demonstrating sudden color changes and errors near the boundaries. This Figure highlights the comparison across performance metrics (MSE, PSNR, SSIM) in both paired and unpaired settings, showcasing the effectiveness of the post-processing strategy in enhancing overall quality and facilitating the adoption of virtual staining technologies in clinical environments. For reproducibility details, refer to Sect 2.5. (TIFF) [file pcbi.1013516.s007.tiff]

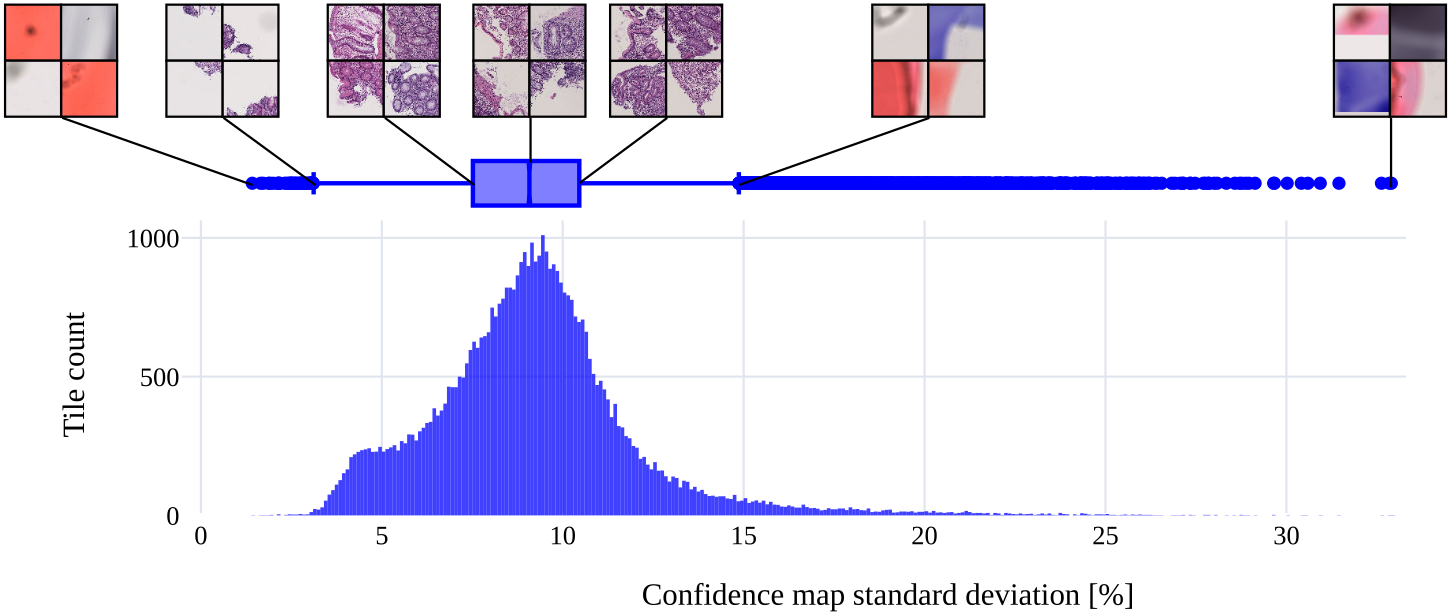

Supplement: S8 Fig — This Figure evaluates the authenticity of 47984 H&E-stained tiles from 2022 authentic WSIs (H&E stained during 20 years of time interval with different scanners) using discriminator confidence maps. The standard deviation of the map is used to assess the authenticity of each tile. The histogram provides pathologists with an empirical tool to determine the acceptable H&E range (e.g., 3.11% to 14.86%), identifying tiles within this range as highly authentic. Tiles outside this range are flagged as outliers, typically due to being background or significantly degraded, indicated by unusually high or consistently low deviations on the confidence maps. These results highlight the discriminator’s ability to identify and quantify tile authenticity, serving as an essential tool for pathologists to exclude unreliable artifacts during the H&E staining and scanning processes. This approach effectively prevents the introduction of substandard images into the multi virtual staining pipeline, thereby reducing the potential error rate in synthetic stains and enhancing the reliability and trustworthiness of generated outputs. For reproducibility details, refer to Sect 2.4. (TIFF) [file pcbi.1013516.s008.tiff]

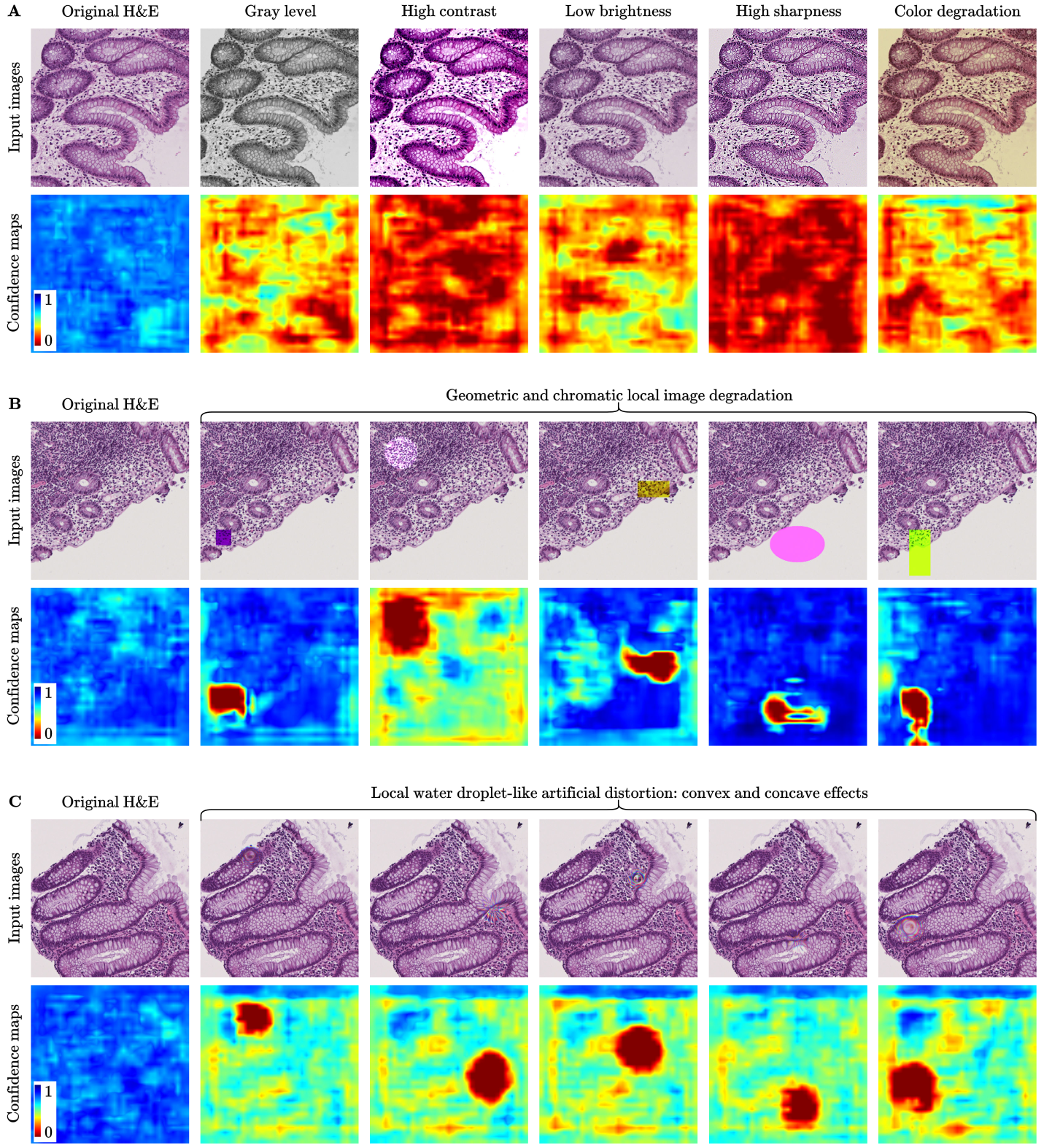

Supplement: S9 Fig — Panels A, B, and C demonstrate the analysis of H&E-stained tiles. In each panel, the top row displays the original H&E tile alongside five degraded versions of the same tile, while the bottom row presents the associated discriminator’s confidence maps. These maps highlight areas of perceptual inconsistency marked in red. Panel A illustrates global degradation potentially caused by issues in chemical staining or scanning errors, such as incorrect staining concentration or scanner configuration problems, and the model successfully identifies such global defects. Panel B shows local contamination possibly due to chemical staining errors or physical artifacts on the scanner, the model pinpointing the locations of the contamination. Panel C depicts artifacts resembling water droplets that can adhere to slides during preparation, potentially causing analysis errors; here, the model indicates the positions of these droplet-like artifacts, thus drawing expert attention to the affected regions. For reproducibility details, refer to Sect 2.4. (TIFF) [file pcbi.1013516.s009.tiff]

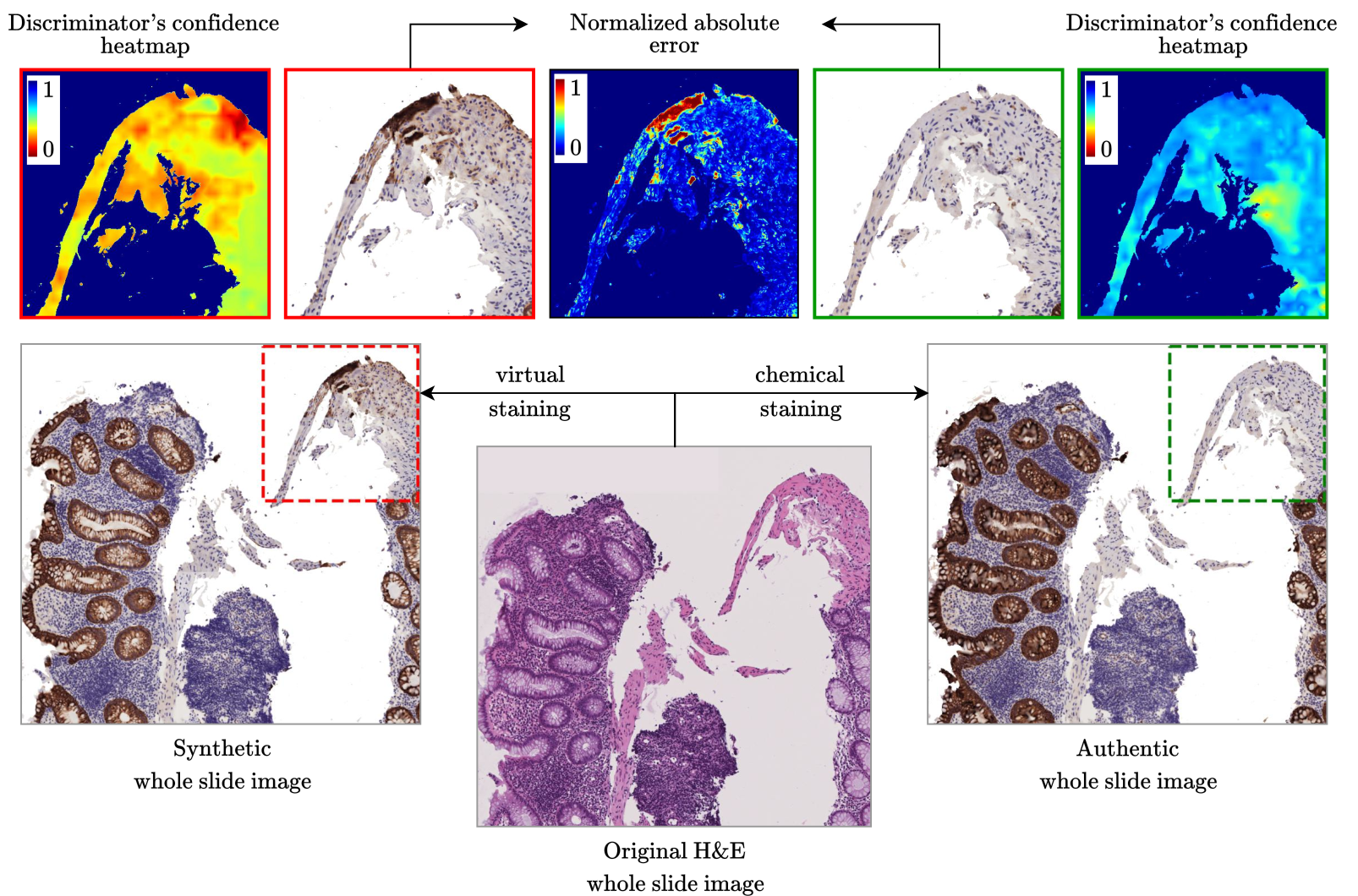

Supplement: S10 Fig — This figure illustrates the effectiveness of discriminator confidence maps in evaluating virtual and authentic stained WSIs. Two tissue sections are shown: one authentically stained and the other virtually stained with an identifiable error. The discriminator response is visualized through heatmaps, where areas of discrepancy are highlighted in red. These highlighted regions correspond to significant deviations from the expected stain appearance, providing pathologists with a pixel-wise confidence score. This visualization helps to determine the need for additional confirmatory chemical staining and to identify critical areas for detailed examination. By quantifying and displaying errors, this tool reinforces the reliability of virtual staining technologies and supports pathologists in making more informed decisions. For reproducibility details, refer to Sect 2.4. (TIFF) [file pcbi.1013516.s010.tiff]

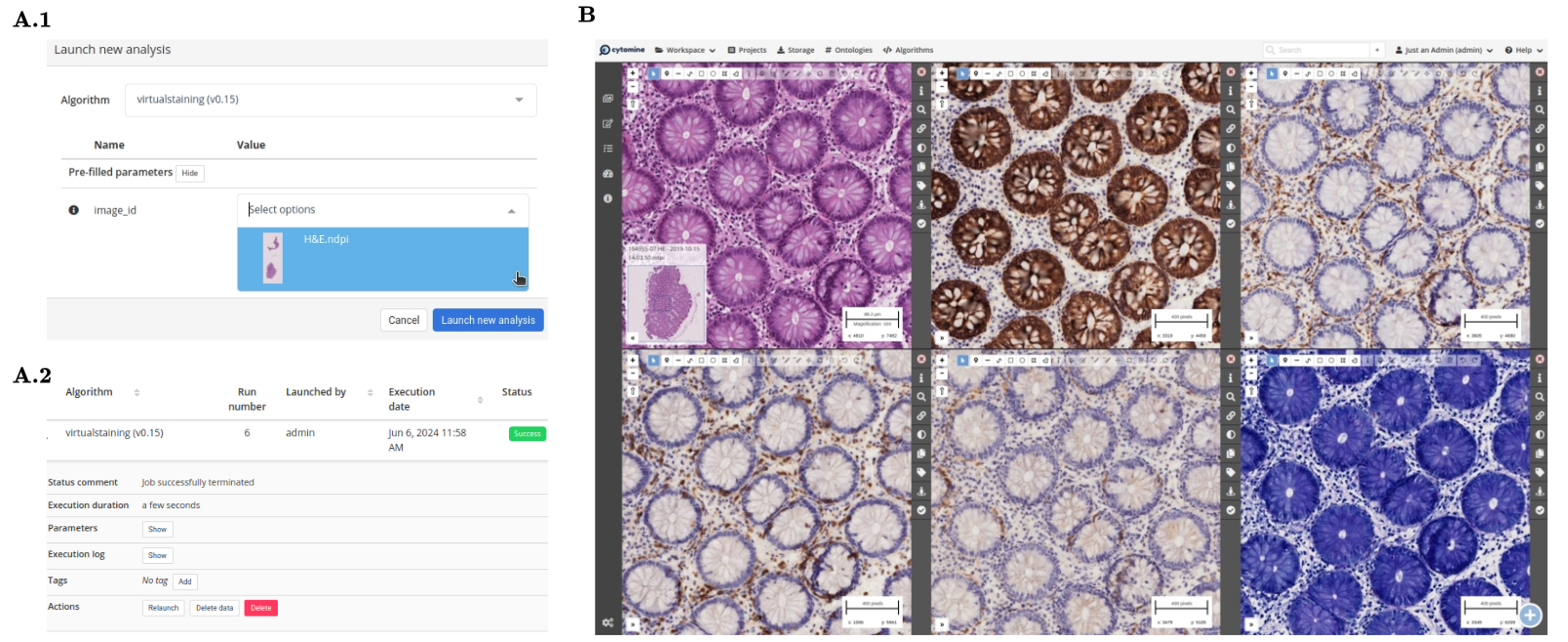

Supplement: S11 Fig — A.1. displays a user interface for selecting the desired H&E WSI and setting the parameters for inference. A.2. illustrates the panel that tracks the progress of the multi virtual staining process (slurm job). B. presents synchronized views of a series of virtually stained slides alongside the original H&E slide (upper left). This Figure demonstrates our dockerized multi virtual staining implementation on the open source Cytomine platform [35] as a use case. Computations are performed on a back-end server (via slurm), with the user only required to upload the H&E slide and initiate the algorithm through the browser. The results are then displayed in a synchronized view, significantly minimizing user effort. For reproducibility details, refer to Sect 2.6. (TIFF) [file pcbi.1013516.s011.tiff]

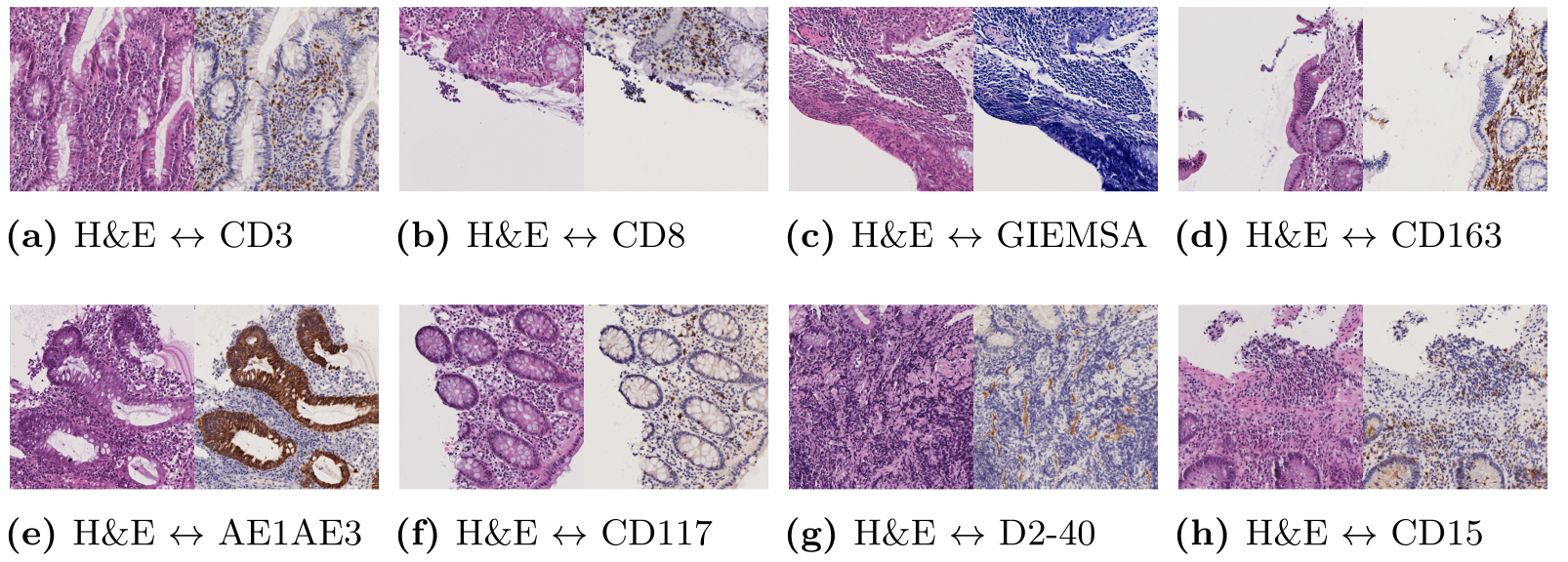

Supplement: S12 Fig — This Figure illustrates the perfect pairing of WSIs from identical tissue sections, which is central to the utility of the dataset in computational pathology research. (TIFF) [file pcbi.1013516.s012.tiff]

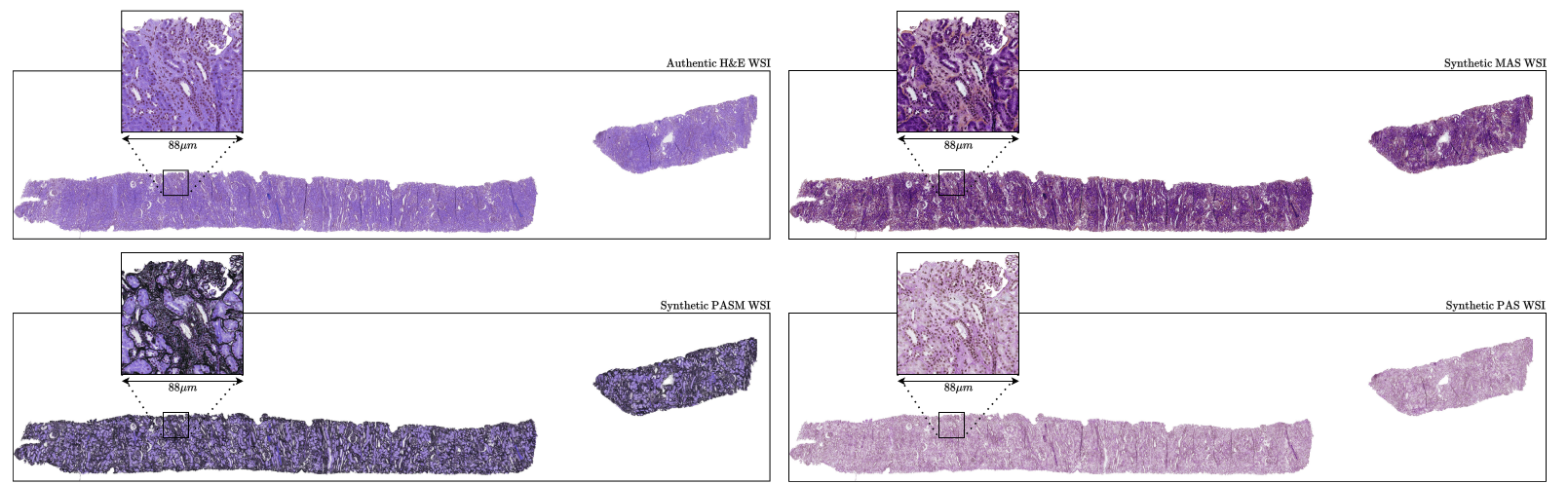

Supplement: S13 Fig — Showing the high-quality synthetic stains generated using our method. (TIFF) [file pcbi.1013516.s013.tiff]

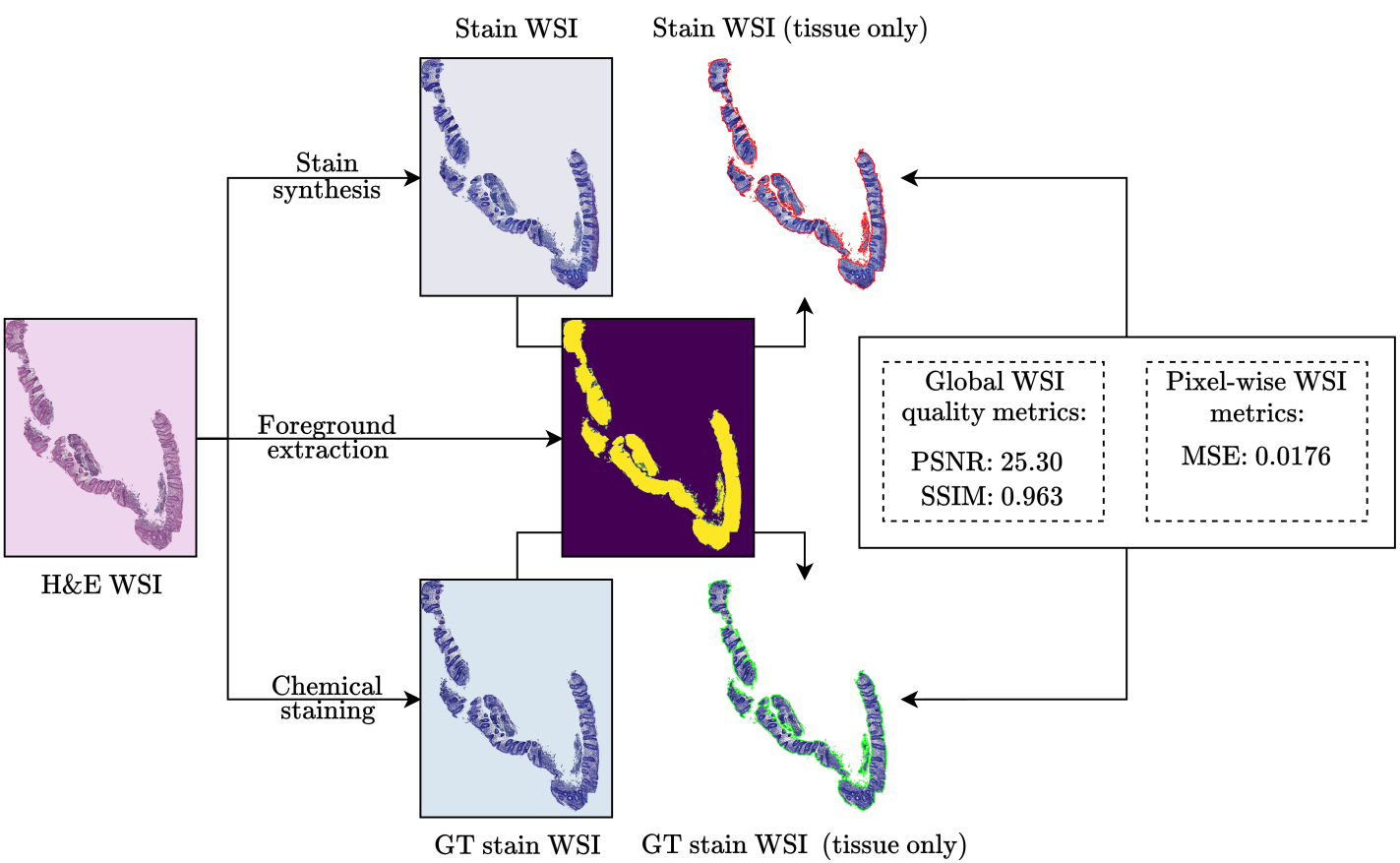

Supplement: S14 Fig — Workflow diagram illustrating the validation process for virtual staining techniques. The process begins with an H&E stained whole slide image (H&E WSI), from which the foreground is extracted. This image undergoes virtual staining to produce the Stain WSI, which is then compared to the chemically stained ground-truth WSI (GT stain WSI). Evaluation metrics include PSNR and SSIM to assess overall image quality and MSE to evaluate pixel-wise accuracy, indicating the effectiveness of the staining simulation. (TIFF) [file pcbi.1013516.s014.tiff]

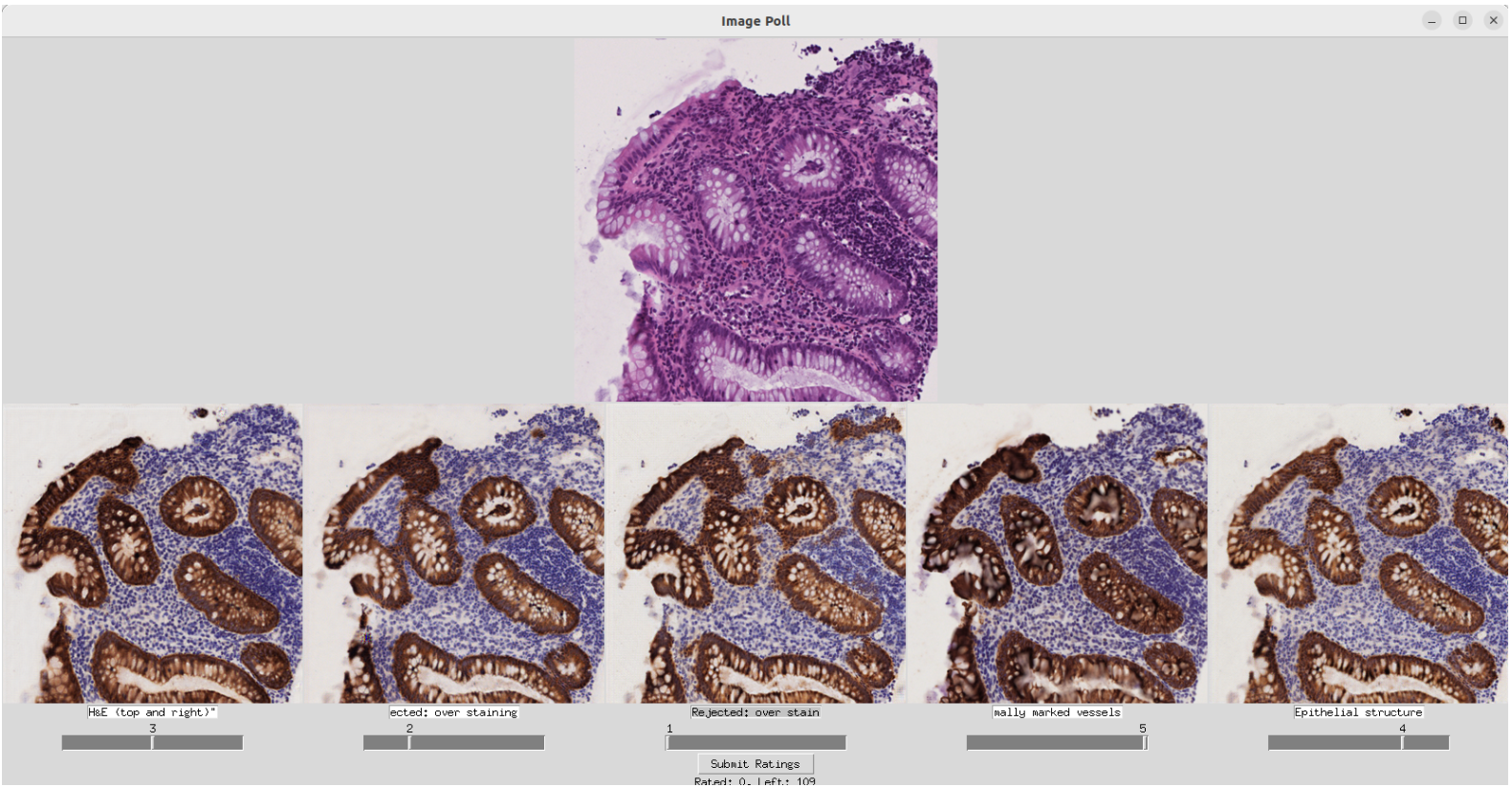

Supplement: S15 Fig — We show the original H&E image at the top, followed by a set of virtual stains in different conditions, including the ground truth randomly shown. The pathologist was asked to rate each image based on the clarity and preservation of morphological details. 1 "worst" 5 "best" with feedback. (TIFF) [file pcbi.1013516.s015.tiff]

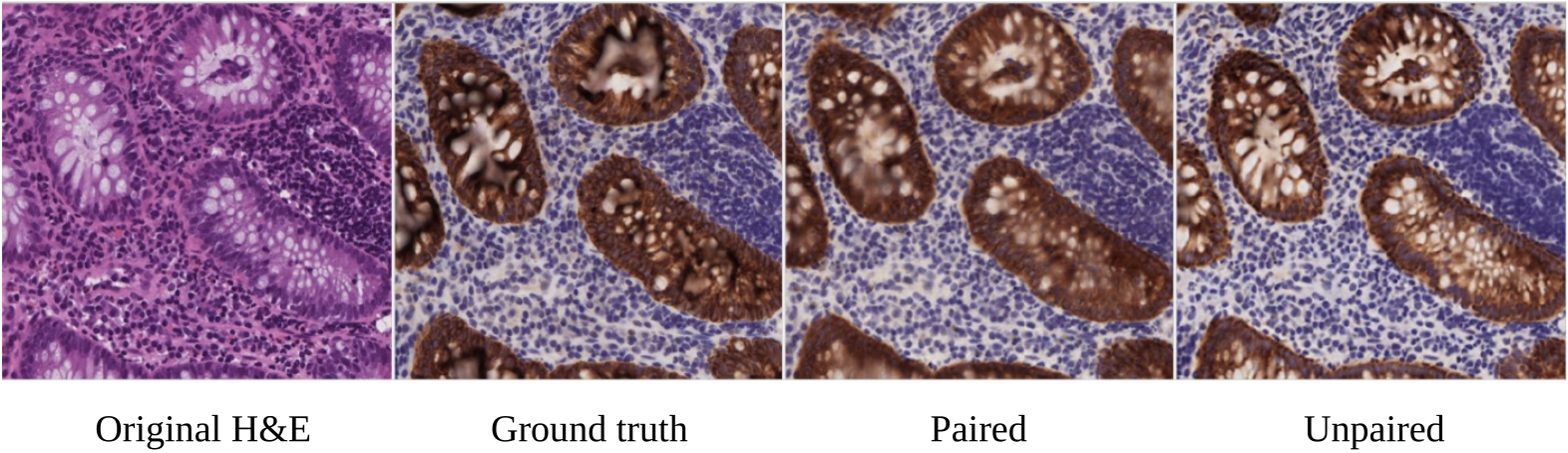

Supplement: S16 Fig — This Figure shows a closer view of the morphological features in the original H&E stain (left) versus virtual ground truth, paired and unpaired stains. The comparison highlights the impact of the water-like blur in chemical stains and its reduction in virtual stains, aiding in the qualitative evaluation by pathologists. (TIFF) [file pcbi.1013516.s016.tiff]

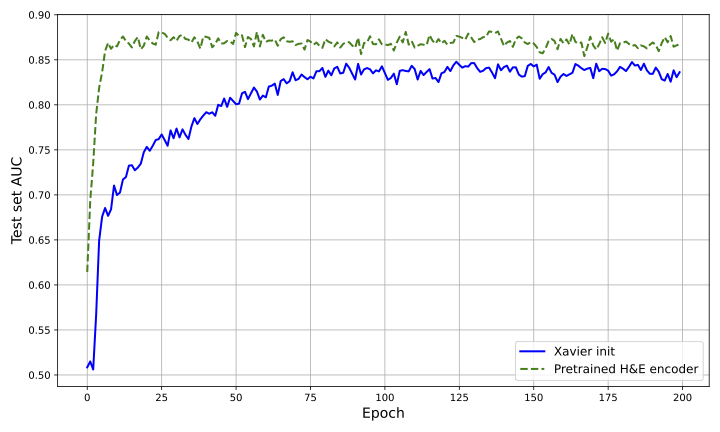

Supplement: S17 Fig — This Figure shows binary cross entropy in the test set during training of a binary classifier from our pretrained H&E encoder and a xavier initialization. The results highlight a faster convergence and better performance when using the pretrained H&E encoder, suggesting the potential of our methods for capturing and transferring protein expression-related features. (TIFF) [file pcbi.1013516.s017.tiff]

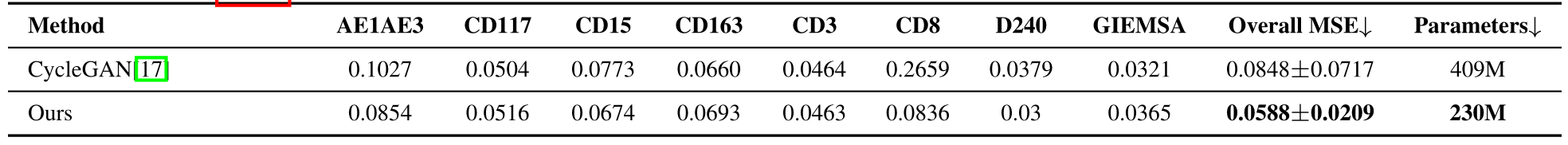

Supplement: S1 Table — This table compares the mean square error (MSE) metrics (mean±std) of synthetic stain generation (unpaired setting) using our unified H&E encoder versus traditional distinct H&E encoders per stain (CycleGAN). The results highlight our method’s superior accuracy and computational efficiency, featuring a significantly reduced number of trainable parameters, thus demonstrating its potential for scalable clinical-effective histopathological applications. For reproducibility details, refer to Sect 2.7.1. (TIFF) [file pcbi.1013516.s018.tiff]

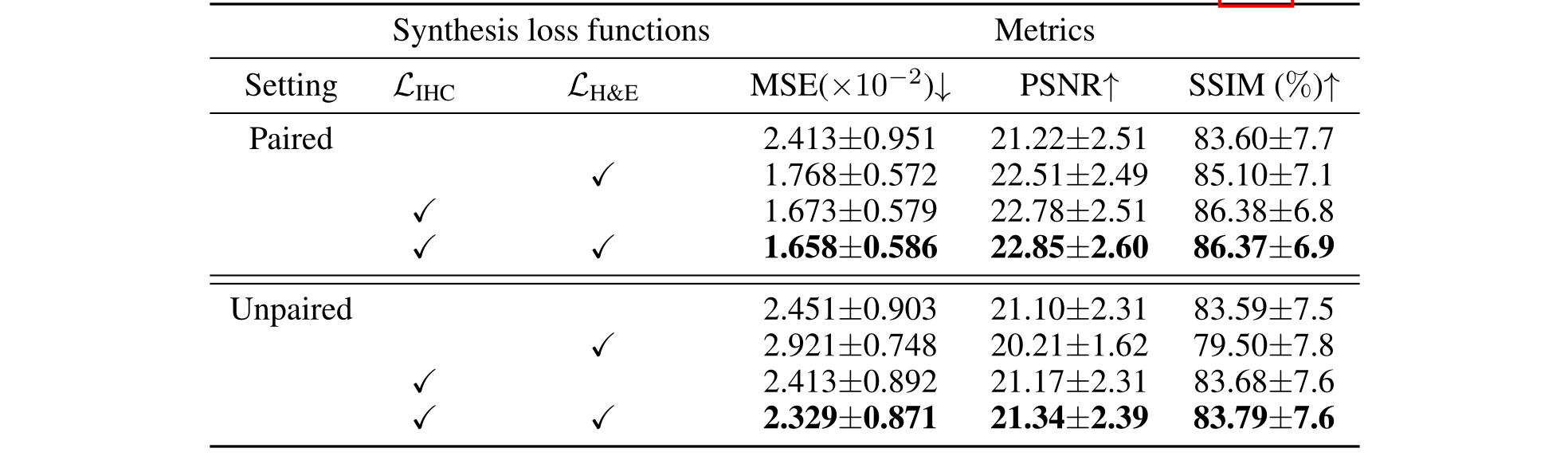

Supplement: S2 Table — Comparative results displayed for paired and unpaired staining settings, quantified by MSE, peak signal-to-noise ratio (PSNR) and structural similarity index (SSIM). For reproducibility details, refer to Sect 2.7.2. (TIFF) [file pcbi.1013516.s019.tiff]

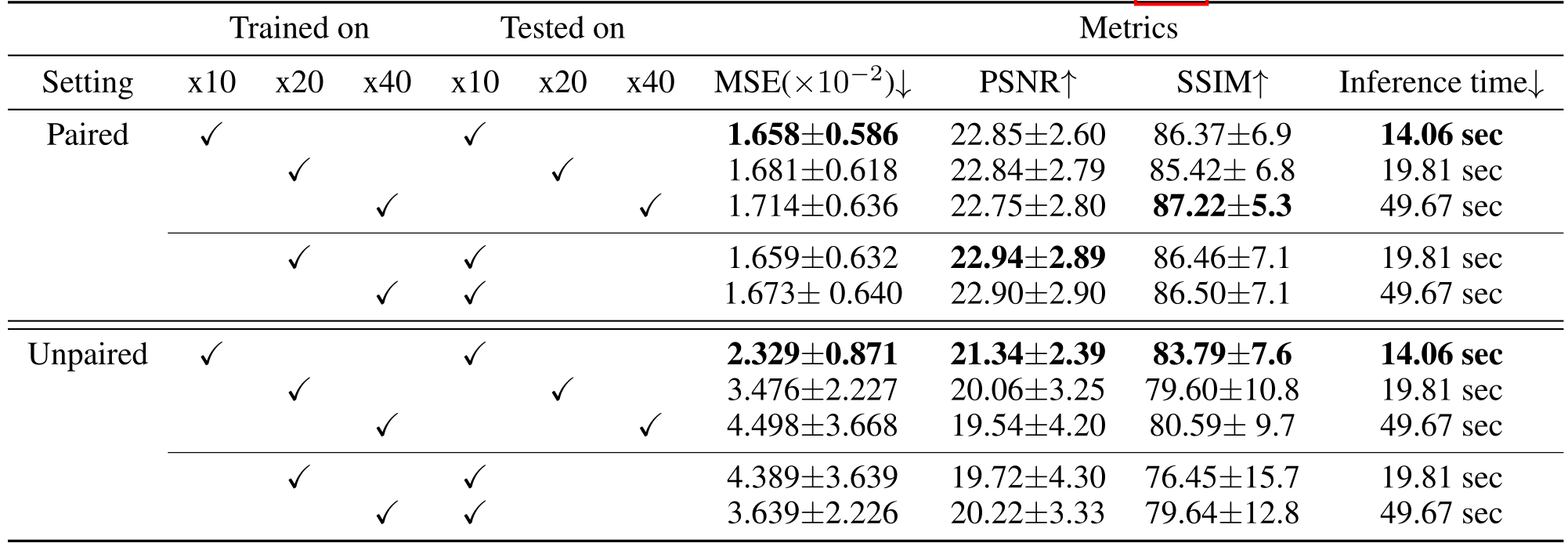

Supplement: S3 Table — This table presents the performance of our modular training approach at different magnifications (x10, x20, x40), where tiles extracted at each magnification were resized to 512 x 512 pixels to ensure a consistent image size for analysis. The models were trained using ℒIHC and ℒH&E loss functions. In the paired setting, no particular magnification preference was observed, indicating uniformity in performance. In contrast, in the unpaired setting, lower magnifications, which provide more contextual information, demonstrated a significant advantage, underscoring the importance of context for effective learning in scenarios lacking direct correspondence between stain types. For reproducibility details, refer to Sect 2.7.3. (TIFF) [file pcbi.1013516.s020.tiff]

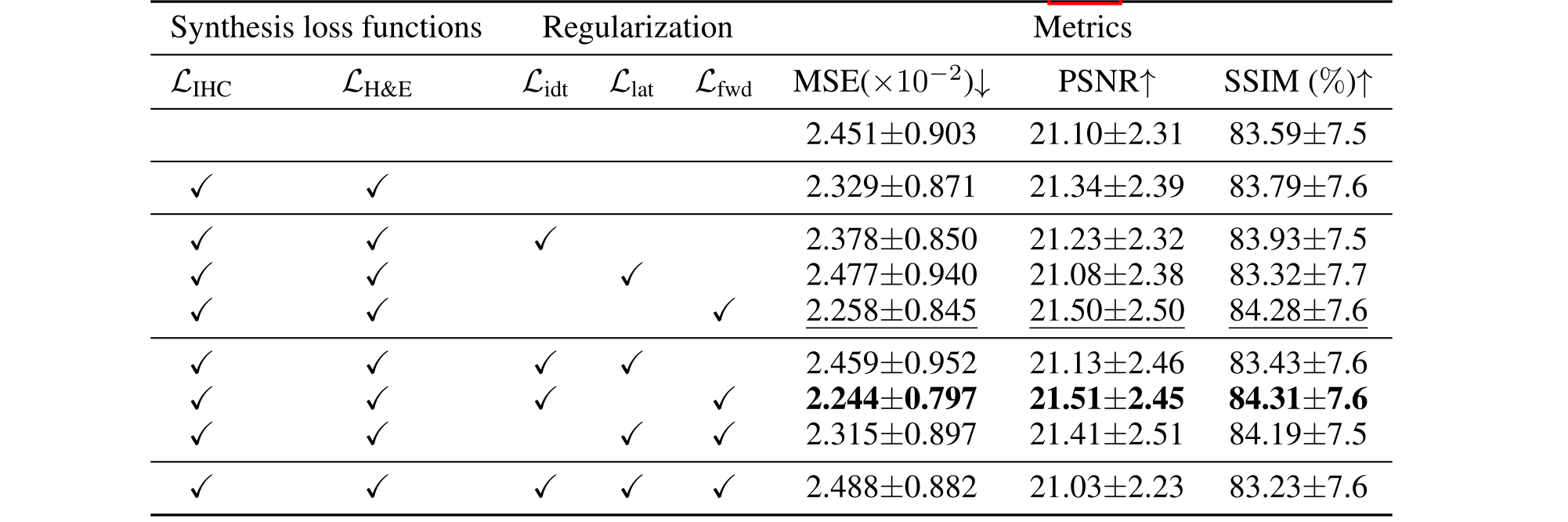

Supplement: S4 Table — This table displays an ablation study of different combinations of synthesis loss functions (ℒIHC, ℒH&E detailed in Sects 2.3.1 and 2.3.2) and regularization methods (ℒidt, ℒlatand ℒfwd detailed in Sect 2.3.2) on the performance metrics MSE, PSNR and SSIM. Each row represents a specific configuration of loss functions, illustrating their impact on the accuracy and quality of virtual staining results. For reproducibility details, refer to Sect 2.7.4. (TIFF) [file pcbi.1013516.s021.tiff]

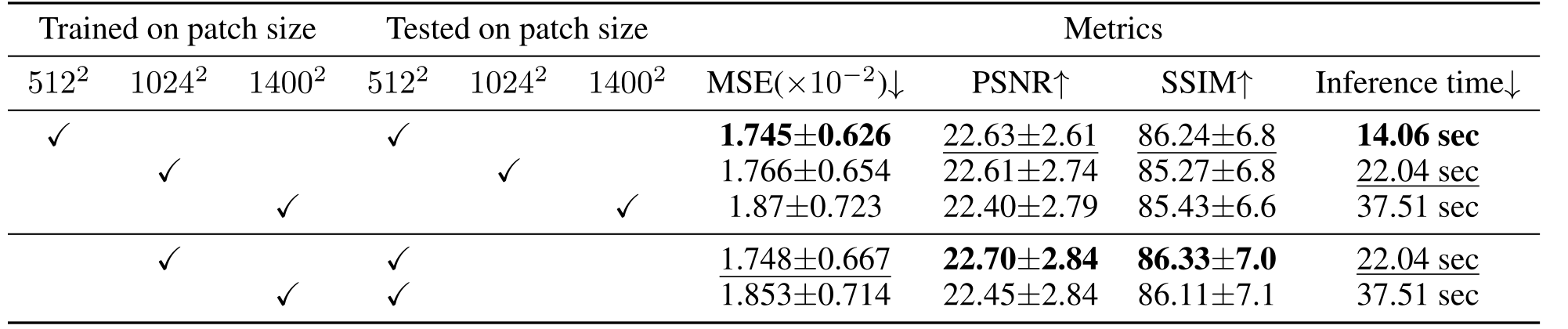

Supplement: S5 Table — This table displays the results of training our virtual staining model on images with eight stains plus H&E at different resolutions. The results demonstrate consistent performance across various densities of pixels. The data highlight our approach’s effective use of advanced GPU resources, emphasizing the scalability of our methodology. (TIFF) [file pcbi.1013516.s022.tiff]

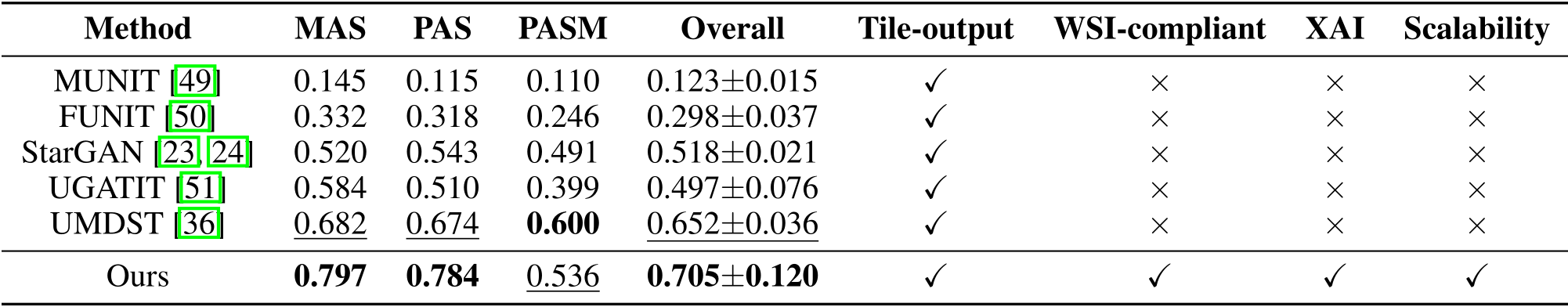

Supplement: S6 Table — This table presents the CSS metrics for various computational methods when applied to human kidney tissue slides stained with H&E, MAS, PAS, and PASM. The performance of each method is evaluated in terms of overall CSS, tile output, WSI-compliant output and evaluation, XAI capabilities, and scalability. The results highlight our method’s superior ability to address the challenges of multi virtual staining, with higher CSS values signifying enhanced preservation of structural similarity across different stains. (TIFF) [file pcbi.1013516.s023.tiff]

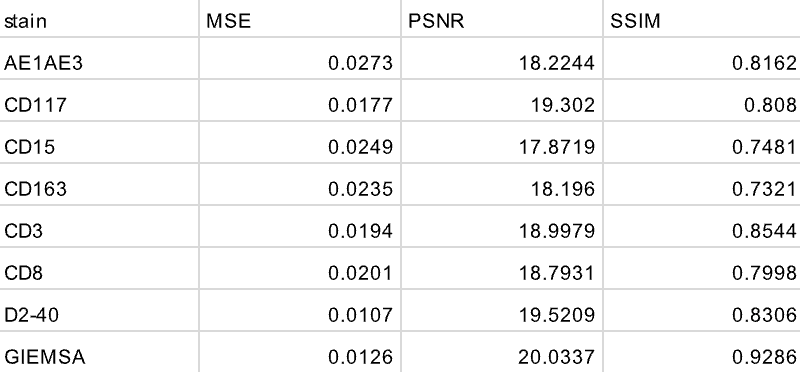

Supplement: S7 Table — This table reports, for each of the eight stainings, the mean squared error (MSE), computed on the masked tissue region, peak signal-to-noise ratio in decibels (PSNR), and structural similarity index (SSIM) computed over the entire tissue region of the WSI. (TIFF) [file pcbi.1013516.s024.tiff]
