# Supplementary material for: Scalable, trustworthy generative model for virtual multi-staining from H&E whole slide images
Source: PLoS Comput Biol. 2025 Oct 21;21(10):e1013516. doi: 10.1371/journal.pcbi.1013516 (PMC12578341; doi:10.1371/journal.pcbi.1013516)
Supplement: S1 Text — (PDF) [file pcbi.1013516.s025.pdf]

S1 Supplementary materials

Generalization to different stains types

The ANHIR [45] dataset includes five sets of high-resolution human kidney tissue slides, each containing four slides of consecutive tissues stained with different types (H&E, MAS, PAS, and PASM staining). Although these slides are structurally similar, they are not pixel-level paired and all are magnified at x40.

According to the experimental setup of UMDST [44], four sets (kidney 1, kidney 2, kidney 3, kidney 4) were used as training sets, and the fifth set (kidney 5) was reserved for testing. From the UMDST protocol, the H&E stained slide from kidney 1 was omitted due to its distinct color variation compared to the other sets. For processing, slides were tiled into 256x256 images with an overlap of 192 pixels.

Our method involved simultaneous training on the three different stains from H&E using four encoders, four decoders, and four discriminators. The training involved 150 000 iterations at a fixed learning rate of 2×10^{-4}, followed by another 150000 iterations with a linearly reducing rate, totaling 300 000 iterations. An Adam optimizer with parameters $\beta_1 = 0.5$ and $\beta_2 = 0.999$, and a batch size of 1 was employed (matching UMDST [44]). Only random flip and random rotation strategies were used for data augmentation. This training was performed on a single NVIDIA A100 80GB GPU. The loss weights were established at $\lambda_{\text{cyc}} = 10$ and $\lambda_{\text{adv}} = 1$, with $\alpha = 0$, $\beta = 0$, and $\mathcal{L}_{\text{H\&E}}$ applied every three iterations. Here, the losses in cycle consistency $\mathcal{L}_{\text{cyc},i}$ from the components of the kidney dataset were summed and averaged. $\mathcal{L}_{\text{idt}} = 0$, $\mathcal{L}_{\text{lat}} = 0$, and $\mathcal{L}_{\text{fwd}} = 0$ were also included in the model.

The Kidney 5 test was conducted as illustrated in S13 Fig, with qualitative results summarized in S6 Table. For benchmarking against state-of-the-art methods, the Contrast Structure Similarity (CSS) metric [44, 47, 48] was utilized, with results also reported in S6 Table.

S6 Table highlights the superior performance and generalization capabilities compared to state-of-the-art methodologies in MAS, PAS, and PASM staining. The slightly lower performance in PASM staining compared to the results claimed by [44] is related to the aim of the method to preserve the H&E morphological features (this improves the CSS metric which is computed between the H&E and the PASM stain). Indeed, PASM staining tends to obscure some morphological features due to its use of black coloration, thus our model learns to do so as well (it is a feature, not a bug). If the goal is to preserve these characteristics (resulting in a less realistic PASM but with all H&E features), a forward loss strategy can be used, as described by [44]. However, this introduces a trade-off between maintaining morphological detail and achieving accurate staining. Furthermore, our approach is scalable during both training and inference and uniquely integrates XAI capabilities, which are not addressed by other methodologies but are critical in a clinical context.

Validation protocol for virtual staining

Quantitative evaluation

To address the inherent limitations of patch-level evaluation in virtual staining, such as restricted contextual information and potential inconsistencies across different tissue regions, we developed an adapted validation protocol. Traditional metrics often fail to capture the nuanced discrepancies that can occur across various regions of a tissue slide, leading to an incomplete assessment of stain quality. In contrast, our protocol incorporates both PSNR and SSIM to comprehensively assess the quality of WSIs. These metrics are crucial for evaluating the fidelity and structural integrity of virtually stained images. Furthermore, the MSE metric is specifically used to provide a quantitative assessment at the tissue pixel level, significantly improving the precision in evaluating staining accuracy.

The use of a paired dataset, where each virtual stain is directly compared to a chemically stained ground-truth counterpart (GT stain WSI), is pivotal. This pairing ensures that each evaluation metric not only measures the error or similarity in isolation, but does so in a context that reflects true biological and clinical scenarios, ensuring the relevance and applicability of the findings.

The refined validation protocol involves several steps. Initially, an H&E stained WSI is processed to extract the foreground, effectively distinguishing the tissue from the background. Subsequent virtual staining algorithms synthesize the stain, producing a WSI stain that is then compared against the ground-truth stain WSI obtained from chemical staining. This comparison is essential to evaluate the performance of virtual staining on entire slides and at the pixel level, as illustrated in S14 Fig. Through these metrics, our protocol addresses critical gaps in existing evaluation methods and sets a clear validation of virtual staining technologies in pathology.

To illustrate the utility of the learned H&E representations for downstream tasks, we conducted an experiment using the HER2 dataset [49], which includes 192 whole slide images (WSIs) from 192 breast cancer patients, stained with H&E and accompanied by expert tumor annotations. The original study demonstrated that HER2-positive and HER2-negative cases could be reliably distinguished using tumor-containing patches. Similar to previous work [49], we extracted 512×512 pixel patches overlapping with the annotated tumor regions (more than 1%). Patients were split into training and testing cohorts, with 80% (148 patients) used for training and 20% (38 patients) for testing. This resulted in a training and validation set of 4,996 patches (2,155 HER2-positive and 2,841 HER2-negative) and a test set of 1,133 patches (616 HER2-positive, 517 HER-2negative). We trained a classifier to predict HER2 status using a ResNet-based architecture initialized from scratch and also using our pretrained H&E encoder. Standard data augmentation strategies were applied during training, including horizontal and vertical flips, grayscale conversion, color jittering, random rotation, and cropping. A learning rate of 0.0001 was used. Trainings with various amounts of epochs was performed (10,20,30,...,190,200) to assess the number with the validation set (1492). Results showed faster convergence when using the pretrained H&E encoder compared with training from scratch (20 and 110 epochs respectively, S17 Fig). Results on test set were also better with AUC 0.877 and 0.840 respectively. These results demonstrate the capability of IHC pretraining to distinguish tissue structures and capture the microenvironment of proteins more effectively in downstream tasks, thus showcasing the added value of our approach for clinical applications in digital pathology.

We further performed a whole-slide per-stain evaluation by computing the mean squared error (MSE), peak signal-to-noise ratio (PSNR) and structural similarity index (SSIM) between each virtually stained WSI and its corresponding chemically stained ground truth. The mean results for all eight markers are reported in S7 Table.

Membrane-associated D2-40 achieved the highest fidelity (MSE = 0.0107; PSNR = 19.52 dB; SSIM = 0.8306), closely followed by the pan-cellular GIEMSA stain (MSE = 0.0126; PSNR = 20.03 dB; SSIM = 0.9286). Cytoplasmic AE1/AE3 exhibited the largest reconstruction error (MSE = 0.0273; PSNR = 18.22 dB; SSIM = 0.8162), reflecting the increased difficulty of modeling filamentous intermediate filament networks. The remaining CD markers showed intermediate performance (e.g. CD3: MSE = 0.0194; PSNR = 19.00 dB; SSIM = 0.8544 versus CD163: MSE = 0.0235; PSNR = 18.20 dB; SSIM = 0.7321), consistent with their membrane-localized expression patterns. These findings indicate that our virtual-staining pipeline performs well on uniform or membrane-restricted targets but is challenged by complex cytoplasmic architectures.

Qualitative evaluation

In our study, we recognize the importance of qualitative evaluation in conjunction with quantitative metrics, particularly from a pathological perspective. Despite using a paired dataset, qualitative evaluation remains crucial to verifying the applicability and precision of our virtual staining techniques from a clinical standpoint.

In S15 Fig, we conducted a poll involving 26 images stained with AE1AE3, where a pathologist was shown the original H&E image alongside virtual staining results. These included images processed through real chemical staining (ground truth) and those generated via our paired and unpaired DL models. The pathologist was instructed to rate the images on a scale from 1 (worst) to 5 (best) and provide feedback.

The results of our study were somewhat counterintuitive. In the assessment of 26 AE1AE3-stained images, ground truth images, which involved actual chemical staining, generally scored lower than those from both the paired and unpaired settings. Specifically, ground-truth images received an average score of 2.69 ± 1.46. In contrast, images from the paired setting, where virtual staining was trained on paired data, scored slightly higher at 3.11 ± 1.63. In particular, the unpaired setting, involving trained virtual staining without paired data, performed the best with an average score of 3.42 ± 1.65. This suggests an unexpected performance trend where virtually generated stains were preferred over actual chemical stains, indicating a discrepancy in quality perception between the traditional and computational methods.

Upon analyzing the pathologists' feedback, a critical observation was made, as illustrated in S16 Fig. It appears that a water-like blur inherent in the chemical staining process tended to obscure the morphological details of the tissue. This issue was less pronounced in the images from the paired and unpaired settings.

In particular, the unpaired model showed superior preservation of morphological features. This is likely because, during training, the model does not directly correlate the H&E images with specific stains, allowing it to learn where to place the stains effectively without replicating the blurring seen in the ground truth. Conversely, the paired model, learning from the blurred ground truth images, tends to reproduce similar artifacts, thus inheriting and replicating these biases. These findings underscore that unpaired training can provide more generalized and unbiased results. Although objective metrics might suggest lower performance compared to paired settings, the qualitative benefits from a pathological perspective are worth noting. The unpaired setting produces visual results that surpass the ground truth (when there is a blur effect), providing enhanced clarity and detail that are crucial for an accurate medical diagnosis.

Testing IDs for crohn's dataset

For reproducibility, the specific slide IDs assigned to the test set for the dataset are designated as follows: AE1AE3 test slides include numbers 9, 13, 19, 21, 22, 24, 26. For CD3, the test slides are 0_0, 0_1, 0_2, 5_0, 5_1, 11. CD8 test slides are numbered 0, 1, 12,

14, 15, 17. CD15 slides identified for testing are 6_0, 6_1, 6_2, 8_0, 8_1, 8_2, 11_0, 11_1, 11_2, 14_0, 14_1, 14_2. CD117 includes test slides 2_0, 2_1, 2_2, 8_0, 8_1, 8_2, 10_0, 10_1, 10_2, 13_0, 13_1, 13_2. CD163 comprises slides 6, 9, 12, 15, 25, 27. D2-40 test slides include 3_0, 3_1, 3_2, 3_3, 7, 9. Lastly, GIEMSA slides for testing are numbered 3_0, 3_1, 3_2, 8_0, 8_1, 8_2, 9_0, 9_1, 9_2, 9_3, 11_0, 11_1.
